# Counting on U training to enhance trusting relationships and mental health literacy among business advisors: protocol for a randomised controlled trial

**DOI:** 10.1186/s12888-022-04034-7

**Published:** 2022-06-15

**Authors:** L. Saxon, S. Bromfield, S. H. Leow-Taylor, C. E. Vega, M. Berk, A. D. LaMontagne, A. J. Martin, M. Mohebbi, K. Nielsen, N. J. Reavley, A. Walker, A. Conway, A. de Silva, K. Memish, A. Rossetto, G. Tanewski, A. Noblet

**Affiliations:** 1 Deakin Business School, Deakin University, Burwood, VIC Australia; 2 The Institute for Mental and Physical Health and Clinical Translation (IMPACT), Food and Mood Centre, School of Medicine, Barwon Health, Deakin University, Burwood, VIC Australia; 3 Orygen, The National Centre of Excellence in Youth Mental Health, Melbourne, VIC Australia; 4 Centre for Youth Mental Health, Florey Institute for Neuroscience and Mental Health, Melbourne, VIC Australia; 5 Department of Psychiatry, The University of Melbourne, Melbourne, VIC Australia; 6 Department of Public Health and Preventive Medicine, Monash University, Melbourne, VIC Australia; 7 Institute for Health Transformation, Deakin University, Burwood, VIC Australia; 8 Tasmanian School of Business and Economics, University of Tasmania, Hobart, TAS Australia; 9 Biostatistics Unit, Faculty of Health, Deakin University, Burwood, VIC Australia; 10 Sheffield University Management School, Sheffield, UK; 11 Centre for Mental Health, Melbourne School of Population and Global Health, The University of Melbourne, Melbourne, VIC Australia; 12 School of Psychology, Deakin University, Geelong, VIC Australia; 13 Institute of Public Accountants, Melbourne, VIC Australia; 14 Research Division, WorkSafe Victoria, Geelong, VIC Australia; 15 School of Public Health and Preventive Medicine, Monash University, Melbourne, VIC Australia; 16 Beyond Blue, Melbourne, VIC Australia; 17 Mental Health First Aid Australia, Parkville, VIC Australia

**Keywords:** Mental health first aid, Depression, Prevention, Mental health conditions, Relationships, Business advisors, SMEs

## Abstract

**Background:**

Financial distress is thought to be a key reason why small-medium enterprise (SME) owners experience higher levels of mental health conditions compared with the broader population. Business advisors who form trusting, high-quality relationships with their SME clients, are therefore well placed to: (1) help prevent/reduce key sources of financial distress, (2) better understand the business and personal needs of their clients and, (3) recognise the signs and symptoms of mental health conditions and encourage help-seeking where appropriate. The aim of this study is to compare the effectiveness of relationship building training (RBT) combined with mental health first aid (MHFA) training for business advisors with MHFA alone, on the financial and mental health of their SME-owner clients.

**Methods:**

This is a single blind, two-arm randomised controlled trial. Participants will be business advisors who provide information, guidance and/or assistance to SME owner clients and are in contact with them at least 3 times a year. The business advisors will invite their SME-owner clients to complete 3 online surveys at baseline, 6- and 12-months. Business advisors will be randomised to one of two conditions, using a 1:1 allocation ratio: (1) MHFA with RBT; or (2) MHFA alone, and complete 3 online surveys at baseline, 2- and 6-months. Primary outcomes will be measured in the business advisors and consist of the quality of the relationship, stigmatizing attitude, confidence to offer mental health first aid, quality of life and provision of mental health first aid. Secondary outcomes will be measured in the SME owners and includes trust in their business advisors, the quality of this relationship, financial wellbeing, financial distress, psychological distress, help-seeking behaviour, and quality of life. To complement the quantitative data, we will include a qualitative process evaluation to examine what contextual factors impacted the reach, effectiveness, adoption, implementation, and maintenance of the training.

**Discussion:**

As there is evidence for the connections between client trust, quality of relationship and financial and mental wellbeing, we hypothesise that the combined RBT and MHFA training will lead to greater improvements in these outcomes in SME owners compared with MHFA alone.

**Trial registration:**

ClinicalTrials.gov: NCT04982094. Retrospectively registered 29/07/2021. The study started in February 2021 and the recruitment is ongoing.

**Supplementary Information:**

The online version contains supplementary material available at 10.1186/s12888-022-04034-7.

## Background

Small to Medium Enterprises (SMEs) account for 99% of all businesses in Australia and at least 95% of enterprises in all OECD countries [1, 2]. They contribute significantly to Australia’s social and economic prosperity, with small businesses employing approximately 4.7 million people and accounting for 41% of total employment [3]. Despite their importance, SME owners face significant ongoing challenges. Externally, these challenges include increasing market volatility, lending restrictions, extensive legislative reform, and rapid technological advancements, while internally they include monitoring cash flow, managing staff, and ensuring the smooth administration of the business [2, 4]. These challenges can generate significant financial pressure and in turn, financial distress (or a lack of financial wellbeing) among SME owners [5].

Financial distress, the SME owner's working conditions (e.g., long hours, burden of responsibility, isolation, obligation to work when sick) and their low rates of help seeking are thought to be why this group experiences higher levels of mental health conditions compared with the broader population [5–11]. The COVID-19 pandemic has also triggered a sharp increase in psychological distress among small business owners, as a recent study commissioned by the Australian Government shows 1 in 3 rated their mental health as fair to poor during the first three months of the pandemic [12]. Developing strategies that can help alleviate the financial distress experienced by SME owners, but at the same time encourage owners to seek help for potential mental health conditions, are therefore critical for protecting and promoting their mental health.

One group that has the potential to address financial distress and act as an intermediary between SME owners and mental health services are business advisors, particularly accountants and bookkeepers. SME owners draw on the technical expertise provided by business advisors on a regular basis, and advisors can often form trusting, long-term relationships with their clients [13]. Intuitively, the formation of these more trusting, longer-term relationships provides the potential for business advisors to better understand the business and personal challenges faced by SME owners, to identify the early signs of mental health conditions and to take action to help alleviate the sources of financial distress.

Mental health literacy programs, such as Mental Health First Aid (MHFA) training, are a key strategy for the early identification of mental health conditions (e.g., depression, anxiety) and are designed to reduce their impact (i.e., secondary, and tertiary prevention) rather than preventing them from occurring in the first instance (i.e., primary prevention) [14–17]. A systematic review has shown that MHFA training can improve knowledge of mental health and the treatments available, recognition of mental health conditions and reduce stigma [18]. This training is popular in the health and human service sectors, and one observational study on financial counsellors has been conducted, however it is not known to what extent their clients received mental health first aid-related advice and support [9, 18–23].

The aim of the current study called Counting on U is to evaluate the effectiveness of relationship building training (RBT) combined with mental health first aid (MHFA) for business advisors, compared with MHFA alone, on the relationship between business advisors and their SME-owner clients, and the financial and mental health wellbeing of their clients [20]. The RBT has been guided by primary prevention principles and aims to equip business advisors with the skills to: (1) form more trusting relationships with their clients so that SME owners feel more comfortable disclosing financial difficulties and/or mental health conditions, (2) better understand the needs of the business and the business owner and thus, (3) prevent/reduce the financial distress experienced by SME owners.

In view of the above, we have formulated a set of hypotheses for testing in the current study. We expect that for business advisors randomised to the RBT and MHFA training compared with MHFA alone: (1) will be in a better position to recognise signs of distress in their SME-owner clients, so the quality of their relationship will be stronger, (2) better understand how financial distress may lead to poor mental health, and decrease any stigmatizing attitudes they may have (e.g., depression is a sign of personal weakness) and, (3) be more confident to provide mental health first aid and the frequency in which they do so, will be greater. SME owners whose advisors undertake the RBT and MHFA, compared with MHFA alone will report: (1) greater trust in their business advisor, (2) the quality of the relationship with their business advisor will be stronger, (3) improved financial wellbeing, psychological distress, and quality of life, and (4) greater help seeking intentions and behaviour. This will be an Australian and New Zealand wide study targeting eligible participants across all regions, urban/regional/rural and states, to ensure SME owner clients such as farmers in drought-stricken areas, to city coffee shop owners, have an opportunity to benefit from the program.

## Methods/design

### Study design

This is a two-arm, single blind, superiority, randomised control trial to assess the effectiveness of the intervention arm (RBT and MHFA), relative to the control arm (MHFA alone) on the respective primary and secondary outcomes for business advisors and their SME-owner clients. Using a mixed-methods approach, we will collect quantitative survey data from business advisors and their SME owner clients and qualitative data from interviews with business advisors, SME-owners, instructors, training vendors, and the accounting and bookkeeper member bodies (Supplementary file 1).

### Recruitment

#### Business advisory professionals

The recruitment of business advisors into the online training program, Counting on U, will be conducted over 18 months via membership of their respective professional organisation (i.e., Chartered Accountants of Australia and New Zealand (CAANZ), Certified Practicing Accountants (CPA) Australia, Institute of Public Accountants (IPA), Institute of Certified Bookkeepers (ICB)). Representatives from each organisation will invite their members via email to participate in the training and research program. The project will also be advertised through Deakin University and professional organisations’ newsletters, websites, and social media pages.

**Fig. 1:**
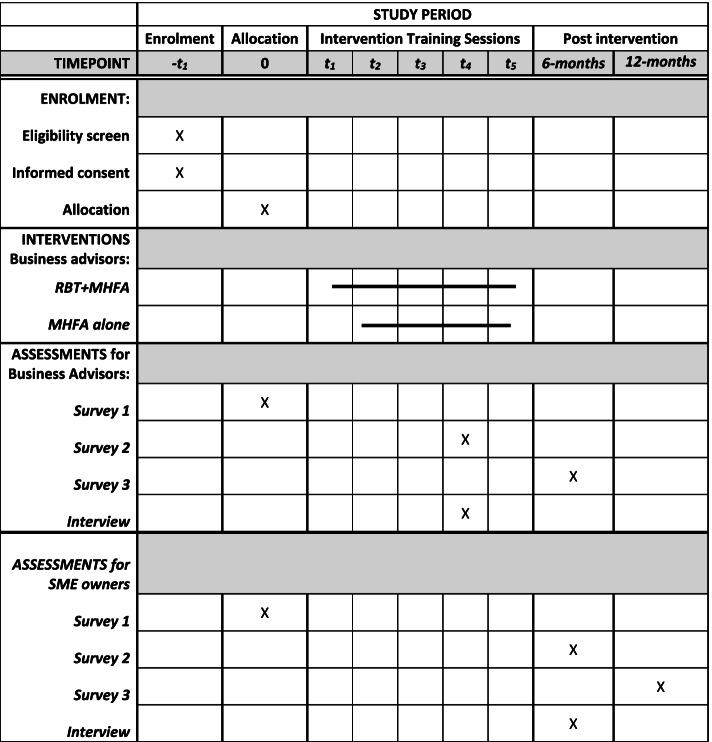
Counting on U schedule of enrolment, interventions, and assessments

**Fig. 2:**
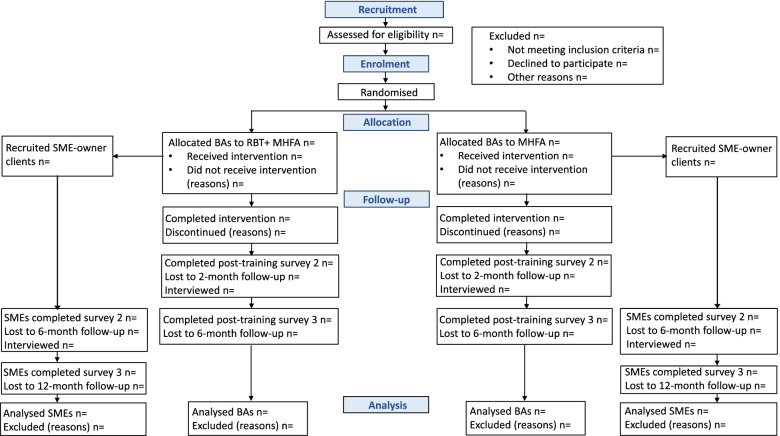
CONSORT diagram showing the enrolment, allocation, quantitative and qualitative data collection time points for the business advisors and their SME-owner clients

Convenience sampling will be used, with all respondents who meet the inclusion criteria offered places in the training program until a total of 984 participants have been recruited. The research team will monitor the participants’ location in urban/regional/rural areas and state, and if a selection bias has occurred, advertising will be targeted to these underrepresented groups. All business advisors will be requested to complete a short online survey at three time-points: baseline, 2 months, and 6 months post-training (equivalent to 1- and 5-months follow-up post-training) (Figs. 1 and 2).

#### SME owners

Business advisors will be asked to invite their SME owner clients to take part in the study to help assess the effectiveness of the training from the perspective of someone who is expected to benefit from it. The SME owner clients will complete 3 online surveys at baseline, 6 and 12 months (or 5- and 11 months follow-up post-training). Business advisors will be requested to send a pre-prepared email to 2–5 of their SME owner clients inviting them to participate (Figs. 1 and 2). However, this is not a mandatory requirement for business advisors.

### Inclusion criteria

The business advisor sample will be selected according to the following inclusion criteria: any qualified business advisor from Australia or New Zealand who provides business-related advice to a small-medium enterprise (SME) client. Business advice refers to the information, guidance and/or assistance provided by an external advisor that either directly or indirectly helps to prevent/reduce the financial pressures experienced by SME owner-clients. The SME owner client must be the owner-manager of the business (i.e., non-operational owners or ‘silent partners’ are not included) and the business needs to employ 1–199 employees. The business advisor must be in contact with their client at least three times a year. The exclusion criteria will be any business advisor who has completed Mental Health First Aid training within the last two years.

Because of our recruitment strategy, eligible participants will be randomised to one of two arms prior to receiving a generic invitation to join the study. They will be informed what study arm they are in, after they have agreed to participate. Thus, participants will only be blind to group allocation prior to providing consent to avoid self-selection bias. Participants must read a plain language statement and sign an online consent form hosted by Deakin University prior to starting (Supplementary file 2). Participants can withdraw at any time by contacting the research team at Deakin University.

### Intervention conditions

#### Relationship building and Mental Health First Aid training

Business advisors randomised to the intervention arm will attend Relationship Building Training (RBT) over a 2-hour live zoom session. The module, which will be delivered by qualified instructors, aims to equip business advisors with the skills they need to enhance trustworthiness, reduce information asymmetry and how to harness their communication skills when having a difficult conversation about finances or mental health with their clients. The RBT module’s content was developed by the principal investigators (AN, GT) and is partially drawn from a post-graduate client relationship building unit taught to accountants and financial planners at Deakin University. This module will be followed by a global, certified, blended Mental Health First Aid (MHFA) training program [20]. The training requires 5 hours of online course work followed by 2 × 2.5-hour live zoom sessions delivered by qualified instructors. MHFA aims to enhance mental health literacy, teach business advisors the skills to identify the signs of mental health conditions, and how to have a conversation with SME owner clients who may need professional help. Participants will also attend 2 × 1-hour Booster sessions 1 and 3-months after completing the training. These booster sessions aim to give participants the opportunity to consolidate their learning, to identify factors that help or hinder the transfer of learning into a change in behaviour and to share experiences on how to maximise their learning. The importance of the business advisors using self-care to protect their own well-being will be reinforced in the RBT and Booster sessions.

#### Mental Health First Aid control group

Participants in the control group will complete the MHFA training alone and attend the two MHFA Booster sessions only.

## Measures

Demographic information including age, sex, and residential data will be collected from the registration forms. The training will have direct effects on the business advisors and in turn, have indirect effects on their SME owner-clients. For this reason, the outcomes measured from business advisors are classed as primary, and for the SME owners they are secondary.

### Primary outcomes for business advisors

#### Quality of the relationship

At each of the data collection points, the quality of the relationship between the business advisor and their SME owner clients will be measured using a Quality of the Relationship Questionnaire. The questions will measure the business advisor’s belief about how much attention they are giving their clients, their awareness about their client’s financial and emotional needs, the quality of the advice they provide and the impact they have on their business. Ten items will be used, four are from the Relationship Flourishing Scale [24] that has a Cronbach’s alpha of 0.93, and six are from a validated newly developed tool by the principal investigators (AN, GT) with factor loadings ranging from 0.81 to 0.87. The Relationship Flourishing Scale has demonstrated acceptable concurrent, convergent and content validities [24]. All items are on a 7-point Likert scale (1 strongly disagree, 7 strongly agree) with a higher total score indicating a higher quality relationship.

#### Stigmatising attitude

The business advisors personal stigmatising attitude will be measured at each time point using eight statements that queries the respondent’s opinion of a person described in a modified vignette (personal stigma) [25]. The statements are based on those developed by Griffiths et al., who also reported on their psychometric properties, showing moderately high convergent validity and acceptable Cronbach alphas of 0.78 to 0.82 [26]. The vignette portrays a 35-year-old business owner who is showing signs of depression. An example of a personal stigma item is: “A problem like John’s is a sign of personal weakness”. All 8 items will be scored on 5-point Likert scale (1 strongly agree, 5 strongly disagree), with a higher score indicating stronger stigmatising attitudes.

Previous work has shown that the 8 items are multi-dimensional and by grouping certain items  they can provide two distinct stigma dimensions “weak-not-sick” and “dangerous/unpredictable” [27, 28]. “Weak-not-sick” suggests the problem portrayed in the vignette is a personal weakness under the control of the person rather than as a medical condition. While “dangerous/unpredictable” characterizes the vignette character as unpredictable and dangerous. These two dimensions can inform whether participants better understand mental health is not a sickness but are still fearful of their behaviour. A factor loading analysis will be conducted to assess which items best load onto each dimension and a total mean and standard deviation score will be generated for each.

#### Confidence to provide Mental Health First Aid

One question will capture the business advisor’s confidence to help a person described in a vignette (see stigmatization) who has a mental health problem. The question will assess their confidence on a 5-point Likert scale (1 not at all confident, 5 extremely confident) and will be included in all three surveys.

#### Provision of Mental Health First Aid

Provision of mental health first aid questions will ask whether the business advisor has talked to a SME owner about their mental health over the past month using a 4-point Likert scale (1 never, 4 many times) [29]. A score of 1 will be given if they answer A Few or Many Times, with higher scores indicating they delivered mental health first aid more often. If they have talked with a SME owner client, they will be asked to indicate how many out of 12 actions they had taken. A score of 1 will be allocated to any action they have answered A Few or Many Times and summing these scores. Business advisors will complete these questions at baseline, 2- and 6-months follow-up.

### Secondary outcomes for SME owners

#### Trust

The degree of trust the SME owner has in their business advisor will be measured using the trust in Business Advisor Questionnaire [30]. This tool assesses three elements of trust: confidence, acting proactively and not exploiting vulnerabilities. In addition, the SME owner’s confidence in the services (i.e., beyond compliance services) offered by the business advisor will be assessed. Eleven questions will be used to assess these elements on a 5-point Likert scale (1 strongly disagree, 5 strongly agree) with a higher total score indicating a more trusting relationship. Factor loadings for these items range from 0.43 to 0.95 and show good reliability, with Cronbach’s alpha of 0.931 to 0.937. The items have demonstrated content validity, i.e., they assess all relevant components of trust [30]. The SME owner will answer these questions at baseline, 6- and 12-months follow-up.

#### Quality of the relationship

At each of the data collection points, the quality of the relationship between the SME owner and their business advisor will be measured using a Quality of the Relationship Questionnaire. The questions will measure the SME owner’s satisfaction on how aware their business advisor is about their wellbeing, whether they can turn to their advisor when things are going badly, and whether they seek their advice on a range of business matters. Six items will be used from a newly developed tool by the principal investigators (AN, GT) with factor loadings of 0.81 to 0.87, and uniqueness scores of 24 to 34%, indicating they are highly relevant to measuring the quality of relationship. All items are on a 7-point Likert scale (1 strongly disagree, 7 strongly agree) with a higher total score indicating a higher quality relationship.

#### Financial wellbeing

The financial wellbeing of the SME owners will be measured at each time point using the Perceived Financial Wellbeing Questionnaire [31]. The SME owners must consider how much they agree with 5 statements such as “I am behind with my finances”. Their answers will be scored on a 5-point Likert scale (1 strongly agree, 5 strongly disagree) and a lower total mean score will indicate a more positive outlook. Perceived financial well-being is a strong predictor of overall well-being and the items have a Cronbach’s alpha of 0.86 to 0.94 [31]. The questionnaire has demonstrated discriminate and incremental validity [31].

#### Financial distress

The financial distress of the SME owners will be measured using the Financial Distress/Financial Wellbeing Scale [32]. This comprises of 8 items designed to assess how positively they view their financial situation, on a score from 1 to 10. A higher mean total score indicates no financial distress/high financial well-being. It has robust internal reliability, with a Cronbach’s alpha of 0.956 indicating that the items consistently yield similar scores. Factor loadings of the 8-items range from 0.83 to 0.93, indicating the measurement of only one dimension and have shown robust content validity [32]. The SME owner’s financial distress will be assessed at baseline, 6- and 12-months follow-up.

#### Psychological distress

Psychological Distress of the SME owners at all time points will be measured using the Kessler 6 (K6) [33]. This broad screening scale assesses individuals for psychological distress as defined as a K6 score ≥ 13. The K6 asks respondents, “In the past four weeks how often did you feel the following: nervous, hopeless, restless, fidgety, worthless, depressed and felt that everything was an effort?” For each question, a value of zero to four is assigned (0 none of the time, 4 all the time), and the total score is summed out of 24 with a higher score indicating greater psychological distress. The scale has demonstrated excellent internal consistent reliability (Cronbach’s alpha = 0.89) and convergent, incremental and divergent validity [33]. 

#### Quality of life

Quality of Life questions will measure the general health of the SME owners at all time points. The Short Form-12 questionnaire (SF-12) produces two summary scores – a mental component score (MCS-12) and a physical component score (PCS-12). This test has a test re-test reliability of 0.76 to 0.89 for the mental (MCS-12) and physical (PCS-12) health components [34]. Both components can discriminate among groups known to differ in their physical and mental conditions, yielding relative validities of 0.63 to 1.07 [34]. The answers are weighted and the results are presented relative to the United States population profile at the time of the original publication in 1994. A higher score for MCS and PCS indicates a better health state.

#### Help Seeking Behaviour

Actual Help Seeking Behaviour questions will assess the SME owners behaviour of actively seeking assistance for any mental health problems at all time points [35]. The scale covers informal and formal assistance, as well as physical and emotional aspects of help-seeking behaviour. The participant is asked to select from a list of people they have gone to for advice or help in the past two-weeks.

Other outcomes will be included in the surveys to test longitudinal models designed by the investigators (AN and GT) that describe how the characteristics of an individual business advisor or SME owner (e.g., resilience) and working conditions (e.g., autonomy, workload) may impact their financial and/or mental wellbeing. These outcomes are described in Supplementary file 3.

### Process evaluation

The second aim of this project is to undertake a comprehensive, research-to-practice process evaluation to ensure that the assessment of Counting on U extends beyond the effectiveness of the interventions [36, 37]. We will draw on the research translation evaluation framework, RE-AIM (i.e., Reach, Effectiveness, Adoption, Implementation, and Maintenance), to explore: the processes used to Reach a nationally representative sample of practising business advisors; how Effective and relevant the intervention is to business advisors and SME owners, and what factors helped or hindered the business advisors to apply what they learnt; what factors influenced the professional member bodies to Adopt the training; whether the instructors Implemented the training in the way it was intended, and; why or why not the professional member bodies decide to Maintain the training as part of their ongoing career professional development program and whether any benefits of the training are Maintained long-term.

To measure these outcomes, field notes and qualitative structured interviews via zoom will be conducted during the training with the business advisors, SME owners, instructors, training vendors, and professional member organization representatives who will assist with the recruitment of business advisors. A risk mitigation-strategy will be designed for the researchers to use in case any interviewee is experiencing poor mental health. To assess intervention fidelity (Implementation), the research team will observe training sessions and complete a fidelity checklist to ensure all topics are covered and that the quality of the teaching is meeting expectations. The research team will observe all 14 instructors deliver the five training sessions (i.e., 1 x RBT, 2 x MHFA, 2 x Booster). The training program will also be monitored through regular weekly meetings between the research team, the instructors, and the training vendor.

### Instructors

The training program will be delivered by 14 instructors who are either accredited MHFA Master Instructors (conducted over 30 MHFA courses) or accredited MHFA Principal Master Instructors (Master Instructors must conduct over 10 MHFA courses in a 12-month period) in Australia and New Zealand. All instructors are accredited to deliver the Blended Online Financial Services Professionals MHFA course. Instructors will also undergo training to deliver the RBT.

### Data management and confidentiality

All participant data will be de-identified using a unique identifier to maintain anonymity. Results will be published in peer-reviewed journals and disseminated to the professional member bodies to communicate with their members. The Deakin University core research team (AN,GT,LS,SB,SHLT,CEV) will have access to the trial data set for the duration of the trial. All data will be kept electronically on a password protected server at Deakin University and securely stored for 5 years with AN and GT. Public access to the full protocol, participant level data-set and statistical code will be available upon request.

### Ethical principles

This study has been approved by Deakin University Human Research Ethics Committee (2020–399). It has been registered with ClinicalTrials.gov with the international standard randomised controlled trial number NCT04982094. Any amendments to the protocol will be approved by the Ethics Committee, will be communicated with ClinicalTrials.gov and any other relevant party (i.e., trainers, participants).

### Trial governance

Appropriate governance structures have been developed to ensure the research team is able to access the advice and guidance provided by each partner organisation that includes WorkSafe Victoria, Beyond Blue, MHFA Australia, The Australian Treasury, CAANZ, CPA, ICB and IPA. A steering committee will be formed consisting of representation from the research team and the eight partner organisations. In addition, the Council of Small Business Owners Australia (COSBOA) has also agreed to be represented on the steering committee. They will play a key role in ensuring this work is grounded in the realities of SME owners (including those who have [or have had] mental health conditions). A small business consultant with high level expertise in working with SME owners and their business advisors will also be employed on a fractional basis (0.1) to work with the steering committee to identify high-impact knowledge translation strategies that can address the needs of both SME owners and their intermediaries. The steering committee will meet on a quarterly basis to oversee trial conduct and monitor data collection. A collaborative decision-making process will used to ensure all members are actively involved in overseeing the project. Investigators will provide an annual report to the Ethics Committee and NHMRC, which will include interim data analysis, any unintended effects of the trial and raise any need to stop the trial.

### Incentives

Business advisors will be incentivised to take part in the training by obtaining continuing professional development (CPD) points that will count towards fulfilling their biennial professional membership requirements, certification as a mental health first aider, and a $30 gift voucher for completing surveys 2 and 3. SME owner participation will be incentivised with a $25 gift card for each survey they complete. One-hundred-dollar gift cards will also be provided to SME owners and business advisors who agree to be interviewed as part of the process evaluation. For SME owners and business advisors who are Australian residents and complete all 3 surveys, they will have a chance to win a $6000 AUD travel voucher via a prize-draw.

### Randomisation

Simple random allocation will be used to ensure the sample size is balanced across the two groups (1:1). Should differential attrition arise part-way through the project, i.e., due to a preference to partake in the intervention, the allocation ratio will be adjusted to compensate for this, i.e., a 1:2 ratio (MHFA+RBT: MHFA). The membership engagement officer at each business advisor’s member organisation (i.e., CAANZ, CPA, IPA, ICB) will conduct the randomisation process by providing eligible participants with a computer-generated unique numeric ID and allocating them to RBT + MHFA or MHFA alone. The participant’s names will be concealed throughout this process. Allocation concealment will be achieved because the member organisations will be unable to predict the unique ID allocated to each participant before they are sorted and randomised to a group. Participants will use their unique ID for completing the surveys, and the researchers will not have access to the business advisor’s contact details only unique ID, group allocation and survey results. Any contact will occur via member bodies or training vendor.

### Sample size and power analysis

The sample size estimation was conducted using Power Analysis and Sample Size (PASS) software (version 15, PASS, NCSS, LLC). The calculations were based on detecting a small effect size (Cohen’s d = 0.20) in the business advisors primary outcome quality of the relationship as measured from an amended Relationship Flourishing Scale [24]. A sample of 786 business advisors randomly allocated (1:1) to the two study arms will achieve 80% power to detect an effect size d = 0.2 in the primary outcome or a mean difference of 1.52 between the two groups. The mean difference of 1.52 was calculated using the results from a validation study of the Relationship Flourishing Scale on 408 participants who reported a mean score of 46.36 and standard deviation of 7.60 [24]. Assuming a 20% attrition rate, we will recruit 984 business advisors. Calculations are based on a two-tailed test and α = 0.05.

### Statistics

A report of the findings will follow CONSORT guidelines. The quantitative analysis will examine whether the magnitude of change in primary and secondary outcomes is different between the SME owners and their business advisors randomly allocated to the MFHA or RBT + MHFA arm (i.e., between-group differential change). Population average models, using a generalised estimated equation (GEE) approach accounting for within-individual repeated measures, will be used to assess for a treatment effect in the primary outcome. The analysis will employ an unstructured correlation structure for the GEE analysis and include a robust covariance matrix estimator, or Sandwich estimator, to provide a consistent estimate of the treatment effect. The GEE approach models the average response of the population rather than modelling the within-subject covariance structure, and unlike a basic regression model, it does not require distributional assumptions. It considers the correlation of within-subject data (longitudinal data) by using all available data for each subject, and it can be used on data with non-normal distributions. The GEE model will contain the fixed effect of intervention allocation and nominal time points as the main effects, and a two-way interaction between intervention allocation and time. The two-way interaction will estimate the effect of differential change from baseline measure in the intervention compared with control group at 2- and 6-months measurements. The GEE model will compare the between-group mean change for business advisors from baseline to 2-months (1-month post-training), baseline to 6-months (4 months post-training), 2-months to 6-months to test the hypothesis (i.e., the two-way interaction between intervention allocation and measurement at 2-months and it’s 95% confidence interval). For SME owners, the between-group differential change will be assessed from baseline to 6-months, baseline to 12-months, and 6-months to 12-months.

In unblinded randomised control trials, participants allocated to the control group who are aware of a more desirable treatment group, may be envious of participants in the treatment group and be less willing to complete surveys [38, 39]. Because of the appeal the new RBT may offer to business advisors, we anticipate that participants in our MHFA alone group may be unmotivated to attend training sessions, complete surveys and ultimately dropout. If such a trend is observed in this study, we may take a pragmatic decision to change the random allocation ratio to allocate more participants to MHFA alone to have equal number of participants in both groups to enhance statistical power. If such a change in the allocation ratio is needed (e.g., from 1:1 to 1:2 to account for differential attrition), the participants will no longer have an equal chance of receiving each of the treatments. Bias could also arise if participants differ in important characteristics, hence the data from these two parts of the trial will be tested for homogeneity. If any significant differences are identified, the results will be analysed separately and then combined, as in a meta-analysis [40].

All analyses will be tested with a significance level of *p* < 0.05 by using the intention to treat principle. Participants classified as lost to follow-up will be compared with the study group for baseline demographic data using ordinary t test and χ2 test for homogeneity. A sensitivity analysis will be conducted to investigate the impact of dropouts on our primary hypothesis. Magnitude of change will be estimated through effect sizes using Cohen’s guidelines, whereby a value of 0.2 denotes a small, 0.5 a medium, and 0.8 a large effect size [41–43]. Data will be entered into Excel using Microsoft Office (version 16.0.1). Professional for Windows XP and StataSE (version 16) software for statistical analysis.

### Qualitative analysis

The structured interviews will be audiotaped and transcribed, then combined with field notes to form a narrative database to be analysed thematically using NVivo (QRS International Version 11) for Windows. A codebook will be created by LS and SB to help identify blocks of texts that represents one of the five RE-AIM constructs. The results of the first round will be compared and any discrepancies will be adjudicated by CEV. A more detailed second round of coding by LS and SB will identify specific themes within these blocks of texts. The results will be discussed by LS, SB and CEV, and a consensus will be reached on the emerging themes. The findings from the qualitative analysis will be used to complement those from the quantitative analysis, so we can better understand what worked for whom in which circumstances.

## Discussion

The aim of this paper is to describe how we will: 1) deliver and evaluate an RCT designed to assess the effectiveness of combining RBT with MHFA for business advisors and their SME owner clients, and 2) conduct a process evaluation to assess what contextual factors impacted the reach, effectiveness, adoption, implementation, and maintenance of the program. To the best of our knowledge, this is the first study to test the effectiveness of using business advisors as a conduit for providing SME owners with mental health support. We believe this approach will ensure SME owners who need help are more likely to receive it.

One of the key strengths of the current research is that the RCT has been developed by a multi-disciplinary team of researchers (spanning the accounting and management sciences, public health and clinical psychiatry), working in conjunction with accounting and bookkeeping member bodies (IPA, CPA, CAANZ, ICB), influential policy makers (Beyond Blue, WorkSafe Victoria, The Treasury) and Australia’s largest provider of MHFA training services (MHFA Australia). The strong partnerships with the professional member bodies are particularly important for ensuring that the trial could be delivered on a sector-wide scale and as part of the CPD training that accountants and bookkeepers are expected to undertake as part of their professional accreditation requirements. The accreditation points gained from the training will help incentivise business advisors to take part and to increase the likelihood that the required number of participants are recruited. In addition, collaborating with member bodies and policy makers will help the authors identify future knowledge translation and sustainability opportunities to address mental wellbeing in the business sector.

The COVID-19 pandemic has increased the likelihood that SME owners will contact their accountant and bookkeeper in heightened states of distress. While this increased demand reinforces the need to equip business advisors with the skills to work with clients experiencing high levels of stress, the anticipated surges in business advisor workloads also represent a key barrier to advisers undertaking the training. To address this, the program has been designed with the business advisors’ operational needs in mind (e.g., fully online, range of training dates offered, incentivised participation). The process evaluation and ongoing dialogue with the professional organisational bodies will also identify any recruitment and retention problems early and to implement appropriate mitigation strategies as soon as possible.

Another strength of this study is using a rigorous RCT design to assess the benefit of offering RBT in combination with MHFA compared with MHFA alone. This design will minimise confounding factors, such as lockdowns during the pandemic, that could have a significant impact on the outcomes. The length of follow-up in business advisors (5-months post training) and SME owners (11-months post training) will also inform whether there are any long-term benefits. Another unique component of this RCT is the recruitment of SME owner clients, who as end-users or beneficiaries of the training, will provide insight into the effectiveness of the training. However, the recruitment of SME owners is expected to be difficult largely because of the busy, all-consuming nature of their role (e.g., long work hours, burden of responsibility). It is for this reason, we introduced gift voucher incentives for SME owners to complete the surveys and to participate in interviews. Another strength of the study is its large sample size predicated on conservative effect size estimates.

To date, the business environment has not been leveraged as a platform for delivering mental health interventions, notwithstanding the major impact of mental health conditions on the sector. The findings from this research will provide important information on the benefits of incorporating a mental health literacy program like MHFA within a more mainstream client relationship building program. If the current research supports the hypothesis that the combined approach leads to more beneficial outcomes than MHFA alone, then the approach could be adopted by other small business intermediaries (e.g., human resource consultants, lawyers, bankers). Similarly, these findings could also be used to inform how business accounting and finance students are trained prior to entering the workforce (i.e., via undergraduate and post-graduate degrees). Finally, the process evaluation will be critical for developing a more detailed understanding of the range of factors that helped or hindered program effectiveness and the success of its roll-out. This information, along with the outcomes of the RCT, can be used to guide efforts to enhance program reach, adoption, implementation, and maintenance in the business sector.

## Supplementary Information

**Additional file 1.** Survey and Interview guides.

**Additional file 2.** Plain Language Statement for business advisors.

**Additional file 3.** Additional Outcomes.

## Data Availability

The data for this study will be made available upon request to George Tanewski: g.tanewski@deakin.edu.au. The research team will make the findings publicly available at national and international conferences, and in peer-reviewed journal publications.

## Abbreviations

CONSORT: Consolidated standards of reporting trials
GEE: Generalised estimated equation
MHFA: Mental health first aid
RCT: Randomised control trial
RE-AIM: Reach, effectiveness, adoption, implementation, maintenance
RBT: Relationship building training
SME: Small-medium entrepreneurs
