# Supplementary material for: Counting on U training to enhance trusting relationships and mental health literacy among business advisors: protocol for a randomised controlled trial
Source: BMC Psychiatry. 2022 Jun 15;22:400. doi: 10.1186/s12888-022-04034-7 (PMC9199223; doi:10.1186/s12888-022-04034-7)
Supplement: Supplementary file 1 — Additional file 1. Survey and Interview guides. [file 12888_2022_4034_MOESM1_ESM.docx]

**Supplementary File 1**

Contents

[Business Advisor Survey 1 1](#_Toc104206093)

[Small-Medium Enterprise Owners Survey 1 24](#_Toc104206094)

[Interview for Business Advisors 43](#_Toc104206095)

[SME Interview 48](#_Toc104206096)

[Trainer Interview Guide 54](#_Toc104206097)

[Training Vendor Interview Guide 57](#_Toc104206098)

[Partner Interview Guide 60](#_Toc104206099)

# Business Advisor Survey 1

Q1
Welcome to the first Counting on U evaluation survey. This survey will assess your understanding and knowledge of mental health, the quality of your relationships with your small enterprise owner clients and your overall wellbeing. It should take less than 10 minutes.  
 
By clicking the ‘Begin Survey’ button below, you are agreeing that you have read the Plain Language Statement (read [this](http://blogs.deakin.edu.au/counting-on-u/wp-content/uploads/sites/392/2021/03/Business-Advisor-PLS-NHMRC-Initial.pdf) for Australian participants OR [this](http://blogs.deakin.edu.au/counting-on-u/wp-content/uploads/sites/392/2021/10/Business-Advisor-PLS-NHMRC-Initial-for-NZ-v3.pdf) for New Zealand participants) and that you are consenting to participate in this survey.
 
Here we go....

End of Block: Introduction Block

Start of Block: Block 1

Q2 Please insert your Counting on U unique ID (refer to the email invite where you were sent the link to this survey):

________________________________________________________________

Q51 Name

________________________________________________________________

Q50 In which country are you currently located?

- Australia (1)
- New Zealand (2)

Q47 What is the postcode of your home address?

________________________________________________________________

Q3 Please describe your profession by selecting one of the following categories:

- A qualified accountant (1)
- A qualified bookkeeper (2)
- A qualified financial planner (3)
- Other: (4) ________________________________________________

Display This Question:

If Please describe your profession by selecting one of the following categories: = A qualified accountant

Q4 How would you describe your accounting practice?

- Sole practitioner (1)
- Small firm with partner(s) (2)
- Medium to large firm (3)
- BIG-4 (4)

Display This Question:

If Please describe your profession by selecting one of the following categories: = A qualified bookkeeper

Or Please describe your profession by selecting one of the following categories: = A qualified financial planner

Q5 How would you describe your bookkeeping or financial planning practice?

- Sole practitioner (1)
- Small firm with partner(s) (2)
- Medium to large firm (3)

Q6 Approximately how many people does your organisation currently employ, including you?

________________________________________________________________

Q7 Are you an owner-manager of the organisation?

- Yes – Full owner (4)
- Yes – Part Owner (5)
- No (6)

Display This Question:

If Are you an owner-manager of the organisation? != No

Q8 How many business partners do you have that are active in the business?

________________________________________________________________

Display This Question:

If Please describe your profession by selecting one of the following categories: = A qualified accountant

Q9 Please provide your best estimate of the relative proportion of the type of services you provide **individually** to a client on a daily basis (should add up to 100%):

Accounting services designed to satisfy regulatory, taxation, banking or other requirements (e.g. Tax return, ASIC Annual Statement Package, Statutory Audit, etc.) : _______ (1)

Business advice services such as planning and performance review of operations, benchmarking, systems reviews, corporate finance and related activities, financial planning, etc. : _______ (2)

Total : ________

Display This Question:

If Please describe your profession by selecting one of the following categories: = A qualified accountant

Q10 Please provide your best estimate of the relative proportion of the type of services your **accounting practice/organisation** provides to clients on a daily basis (should add up to 100%):

Accounting services designed to satisfy regulatory, taxation, banking or other requirements (e.g. Tax return, ASIC Annual Statement Package, Statutory Audit, etc.) : _______ (1)

Business advice services such as planning and performance review of operations, benchmarking, systems reviews, corporate finance and related activities, financial planning, etc. : _______ (2)

Total : ________

Q11 In what year did you become a qualified business advisor (eg. accountant/ bookkeeper/ counsellor/ coach/ financial planner)?

________________________________________________________________

Q12 How many hours do you work on average per week?

________________________________________________________________

Q13 Highest level of education

▼ Postgraduate Degree (1) ... Year 11 or below (includes Certificate I/II) (7)

Display This Question:

If Please describe your profession by selecting one of the following categories: = A qualified financial planner

Q14 What gender do you identify with?

- Female (1)
- Male (2)
- Other (please specify) (3) ________________________________________________

Display This Question:

If Please describe your profession by selecting one of the following categories: = A qualified financial planner

Q15 Age

________________________________________________________________

End of Block: Block 1

Start of Block: Block 2

Q16 The next section contains statements about health problems. Please indicate whether you agree or disagree with each statement, or if you don’t know.

|  | Agree (1) | Disagree (2) | Don't know (3) |
| --- | --- | --- | --- |
| Half of all people who experience a mental illness have their first episode by age 18. (1) |  |  |  |
| Depressive disorders are the most prevalent mental illness in the Australian population. (2) |  |  |  |
| If a person who is depressed does not want to seek professional help, it is important to force them to if you can. (3) |  |  |  |
| Exercise can help relieve depression. (4) |  |  |  |
| Recovery from anxiety disorders requires facing situations which are anxiety provoking. (5) |  |  |  |
| Antidepressant medications can be an effective treatment for most anxiety disorders. (6) |  |  |  |
| When interacting with a person with psychosis, it is best not to offer them choices of how you can help them because it could add to their confusion. (7) |  |  |  |
| A person with a psychotic illness is less likely to relapse if they have a good relationship with their family. (8) |  |  |  |
| A good way to help a person with a drug or alcohol problem is to let them know that you strongly disapprove of their substance use. (9) |  |  |  |
| People with mental illnesses are much more likely to be smokers. (10) |  |  |  |
| It is not a good idea to ask someone if they are feeling suicidal in case you put the idea in their head. (11) |  |  |  |
| If a person is cutting themselves to cope with emotional distress, you should avoid expressing a strong negative reaction to the self-injury. (12) |  |  |  |
| It is best to get someone having a panic attack to breathe into a paper bag. (13) |  |  |  |
| If someone has a traumatic experience, it is best to make them talk about it as soon as possible. (14) |  |  |  |
| It is best not to try to reason with a person having delusions. (15) |  |  |  |
| If a person is intoxicated with alcohol, it is not possible to make them sober up more quickly by giving them strong coffee, a cold shower or taking them for a walk. (16) |  |  |  |
| If a person becomes unconscious after taking drugs, it is best to lie them on their side rather than on their back. (17) |  |  |  |
| If a person with a mental illness becomes aggressive, they will generally calm down if spoken to firmly. (18) |  |  |  |

End of Block: Block 2

Start of Block: Block 3

Q17 John is a 35-year-old small business owner who has been feeling unusually sad and miserable for the last few weeks. He is tired all the time and has trouble sleeping at night. John doesn’t feel like eating and has lost weight. He can’t keep his mind on his business and he’s been slow in paying suppliers, processing invoices, and completing other day-to-day tasks. He puts off making any decisions and isn’t as engaged when talking to his staff or customers. John has recently lost two major customers because of a lack of care and engagement. His staff and family are very concerned about him.

|  | Strongly disagree (1) | Disagree (2) | Neutral (3) | Agree (4) | Strongly agree (5) |
| --- | --- | --- | --- | --- | --- |
| A problem like John’s is a sign of personal weakness. (1) |  |  |  |  |  |
| People with a problem like John’s are dangerous. (2) |  |  |  |  |  |
| If I had a problem like John’s, I would not tell anyone. (3) |  |  |  |  |  |
| People with a problem like John’s could snap out of it if they wanted. (4) |  |  |  |  |  |
| John’s problem is not a real medical illness. (5) |  |  |  |  |  |
| It is best to avoid people with a problem like John’s so that you don’t develop this problem yourself. (6) |  |  |  |  |  |
| People with a problem like John’s are unpredictable. (7) |  |  |  |  |  |
| I would not employ someone if I knew they had a problem like John’s. (8) |  |  |  |  |  |

Q18

|  | Not at all confident (1) | A little confident (2) | Moderately Confident (3) | Quite Confident (4) | Extremely Confident (5) |
| --- | --- | --- | --- | --- | --- |
| How confident would you be in your ability to help John? (1) |  |  |  |  |  |

End of Block: Block 3

Start of Block: Block 4

Q19

|  | Never (1) | Once (2) | Occasionally  (a few times) (3) | Frequently (4) |
| --- | --- | --- | --- | --- |
| How often have you talked to one or more of your clients about their mental health problems over the past month? (1) |  |  |  |  |

Display This Question:

If = Never

Q46 For what reason have you not talked to your clients about their mental health problems?

- Clients appear to be okay (1)
- No time to discuss personal struggles (2)
- I don't feel comfortable doing so (3)
- Other reason: (4) ________________________________________________

Display This Question:

If != Never

Q20 If you had talked with your clients about their mental health problems over the past month, please indicate which of the following actions you have taken (select all that apply):

|  | Never (1) | Once (2) | A few times (3) | Many times (4) |
| --- | --- | --- | --- | --- |
| Spent time listening to their problem (1) |  |  |  |  |
| Helped to calm them down (2) |  |  |  |  |
| Talked to them about suicidal thoughts (3) |  |  |  |  |
| Recommended they seek professional help (4) |  |  |  |  |
| Encouraged self-help strategies (5) |  |  |  |  |
| Gave them information about local services (7) |  |  |  |  |
| Made an appointment for them with services (8) |  |  |  |  |
| Referred them to websites about their problem (9) |  |  |  |  |
| Enlisted others to help (10) |  |  |  |  |
| Called crisis services to attend (11) |  |  |  |  |
| Helped deal with a business-related issue contributing to the problem (12) |  |  |  |  |

Display This Question:

If != Never

Q21 Did you do anything else?

________________________________________________________________

Q22 How easy is it for you to talk to the following people?

|  | Very difficult  1 (1) | Difficult  2 (2) | Neutral  3 (3) | Easy  4 (4) | Very easy  5 (5) |
| --- | --- | --- | --- | --- | --- |
| Clients (1) |  |  |  |  |  |
| Family and friends (2) |  |  |  |  |  |
| Colleagues and peers (3) |  |  |  |  |  |

Q23 To what extent are the following people willing to listen to your problems?

|  | Never  1 (1) | Rarely  2 (2) | Occasionally/Sometimes  3 (3) | A moderate amount  4 (4) | A great deal  5 (5) |
| --- | --- | --- | --- | --- | --- |
| Clients (1) |  |  |  |  |  |
| Family and friends (2) |  |  |  |  |  |
| Colleagues and peers (3) |  |  |  |  |  |

End of Block: Block 4

Start of Block: Block 5

Q24 How do you perceive your relationship with your clients?

|  | Strongly disagree (1) | Disagree (2) | Somewhat disagree (3) | Neutral (4) | Somewhat agree (5) | Agree (6) | Strongly agree (7) |
| --- | --- | --- | --- | --- | --- | --- | --- |
| My clients regularly seek out business advice from me. (1) |  |  |  |  |  |  |  |
| My clients can turn to me when things are going badly in the business. (2) |  |  |  |  |  |  |  |
| I know when my clients are doing it tough. (3) |  |  |  |  |  |  |  |
| I check in with my clients to see how they are doing. (4) |  |  |  |  |  |  |  |
| I provide my clients with advice on a range of business matters such as strategic planning, risk management and staffing matters. (5) |  |  |  |  |  |  |  |
| My clients come to me for all of their required financial administration. (6) |  |  |  |  |  |  |  |
| My clients have more success with their important goals because of my help. (7) |  |  |  |  |  |  |  |
| I have helped my clients grow in ways that they could not have done on their own. (8) |  |  |  |  |  |  |  |
| Talking with my clients helps them see things in new ways. (9) |  |  |  |  |  |  |  |
| I show an interest in things that are important to my clients. (10) |  |  |  |  |  |  |  |

End of Block: Block 5

Start of Block: Block 6

Q25  In general, would you say your health is:

|  | Excellent (1) | Very good (2) | Good (3) | Fair (4) | Poor (5) |
| --- | --- | --- | --- | --- | --- |
| (6) |  |  |  |  |  |

Q26 The following two questions are about activities you might do during a typical day. Does **YOUR HEALTH NOW LIMIT YOU** in these activities? If so, how much?

|  | Yes, Limited A Lot (1) | Yes, Limited A Little (2) | No, Not Limited At All (3) |
| --- | --- | --- | --- |
| MODERATE ACTIVITIES, such as moving a table, pushing a vacuum cleaner, bowling, or playing golf: (1) |  |  |  |
| Climbing SEVERAL flights of stairs: (2) |  |  |  |

Q27 During the **PAST 4 WEEKS** have you had any of the following problems with your work or other regular activities **AS A RESULT OF YOUR PHYSICAL HEALTH?**

|  | Yes (1) | No (2) |
| --- | --- | --- |
| **ACCOMPLISHED LESS** than you would like: (1) |  |  |
| Were limited in the **KIND** of work or other activities: (2) |  |  |

Q28 During the **PAST 4 WEEKS**, were you limited in the kind of work you do or other regular activities **AS A RESULT OF ANY EMOTIONAL PROBLEMS** (such as feeling depressed or anxious)?

|  | Yes (1) | No (2) |
| --- | --- | --- |
| **ACCOMPLISHED LESS** than you would like: (1) |  |  |
| Didn’t do work or other activities as **CAREFULLY** as usual: (2) |  |  |

Q29 During the **PAST 4 WEEKS**, how much did **PAIN** interfere with your normal work (including both work outside the home and housework)?

|  | Not at all (1) | A little bit (2) | Moderately (3) | Quite a bit (4) | Extremely (5) |
| --- | --- | --- | --- | --- | --- |
| (1) |  |  |  |  |  |

Q30 The next three questions are about how you feel and how things have been **DURING THE PAST 4 WEEKS**. For each question, please give the one answer that comes closest to the way you have been feeling. How much of the time during the **PAST 4 WEEKS** –

|  | All of the Time (1) | Most of the Time (2) | A good bit of the Time (3) | Some of the Time (4) | A Little of the Time (5) | None of the Time (6) |
| --- | --- | --- | --- | --- | --- | --- |
| Have you felt calm and peaceful? (1) |  |  |  |  |  |  |
| Did you have a lot of energy? (2) |  |  |  |  |  |  |
| Have you felt downhearted and blue? (3) |  |  |  |  |  |  |

Q31 During the **PAST 4 WEEKS**, how much of the time has your **PHYSICAL HEALTH OR EMOTIONAL PROBLEMS** interfered with your social activities (like visiting with friends, relatives, etc.)?

|  | All of the time (1) | Most of the Time (2) | A Good Bit of the Time (3) | Some of the Time (4) | A Little of the Time (5) | None of the Time (6) |
| --- | --- | --- | --- | --- | --- | --- |
| (1) |  |  |  |  |  |  |

End of Block: Block 6

Start of Block: Block 7

Q32 In the past four weeks, how often did you feel….

|  | None of the time (1) | A little of the time (2) | Some of the time (3) | Most of the time (4) | All of the time (5) |
| --- | --- | --- | --- | --- | --- |
| Nervous (1) |  |  |  |  |  |
| Hopeless (2) |  |  |  |  |  |
| Restless or fidgety (3) |  |  |  |  |  |
| That everything was an effort (4) |  |  |  |  |  |
| So depressed that nothing could cheer you up (5) |  |  |  |  |  |
| Worthless (6) |  |  |  |  |  |

Q33

|  | Not at all (23) | Not much (24) | A moderate amount (25) | To some extent (26) | A great extent (27) |
| --- | --- | --- | --- | --- | --- |
| There is not enough time to perform my role. (1) |  |  |  |  |  |
| There is an excessive amount of work in my role. (2) |  |  |  |  |  |
| I experience pressure in my role. (3) |  |  |  |  |  |

Q34

|  | Never/hardly (1) | Seldom (2) | Sometimes (3) | Often (4) | Always (5) |
| --- | --- | --- | --- | --- | --- |
| Does your work put you in emotionally disturbing situations? (1) |  |  |  |  |  |
| Do you have to relate to other people’s personal problems as part of your work? (2) |  |  |  |  |  |

Q35

|  | To a very small extent (1) | To a small extent (2) | Somewhat (3) | To a large extent (4) | To a very large extent (5) |
| --- | --- | --- | --- | --- | --- |
| Is your work emotionally demanding? (1) |  |  |  |  |  |
| Do you get emotionally involved in your work? (2) |  |  |  |  |  |

Q36  How much control do you have over your work?

|  | Strongly disagree (1) | Disagree (2) | Neutral (3) | Agree (4) | Strongly agree (5) |
| --- | --- | --- | --- | --- | --- |
| I have flexibility in the execution of my work. (1) |  |  |  |  |  |
| I have control over how my work is carried out. (2) |  |  |  |  |  |
| I can participate in decision making regarding my work. (3) |  |  |  |  |  |

End of Block: Block 7

Start of Block: Block 8

Q37 Tick one or more of the following who you have gone to for advice or help in the past 2 weeks for a personal or emotional problem.

- Partner (e.g., spouse, boyfriend or girlfriend) (1)
- Friend (not related to you) (2)
- Parent (3)
- Other relative/family member (4)
- Mental health professional (e.g., counsellor, psychologist, psychiatrist) (5)
- Phone help line (e.g., Lifeline, Beyond Blue) (6)
- GP (7)
- Someone else not listed above (8) ________________________________________________
- I have not sought help from anyone for my problem (10)
- I have not needed advice or help. (11)

Q45 Are you happy for us to contact you at a later date to explore some of your answers in more detail?

- Yes (1)
- Maybe (2)
- No (3)

# Small-Medium Enterprise Owners Survey 1

Q1   Thank you for your interest in helping to evaluate Counting on U, a new training and research program for business advisers like accountants, bookkeepers, financial planners and coaches.

Q2 Please enter the unique ID which was provided in your email invitation

________________________________________________________________

Q3 Are you a SME owner-manager who employs between 1-199 people, including yourself?

- Yes (1)
- No (2)

Q4 Do you have contact with your business adviser at least 3 times a year? *(NB: business adviser refers to the person who invited you to participate in this research study)*

- Yes (1)
- No (2)

Q5 Please provide your consent to the following:

|  | (1) |
| --- | --- |
| I have read and understand the attached Plain Language Statement. (2) |  |
| I freely agree to participate in this project according to the conditions outlined in the Plain Language Statement. (1) |  |
| The researchers has agreed not to reveal my identity and personal details, including where information about this project is published, or presented in any public form. (3) |  |

Q6 Please provide your name & email address so we can send you a $25 online gift voucher for every survey you complete (if you are the first 900 SME business owners who agree to participate in this research), as well as future project survey links. Once you've entered your details and click the 'Next' arrow, you'll be directed to the first project survey questions.

Q7 Name

________________________________________________________________

Q8 Email address

________________________________________________________________

Q9 **Counting on U SME Survey 1**

 What is the length of your business relationship with your business adviser? (in years)

________________________________________________________________

| 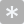 |
| --- |

Q10 Please enter your age

________________________________________________________________

Q11 What gender do you most identify with?

- Male (2)
- Female (3)
- Other (4)

Q70 In which country are you located?

- Australia (2)
- New Zealand (3)

Q12 What industry does your SME business operate within? (choose the one that best represents your business. If none fit, please select 'Miscellaneous')

▼ Aged care industry (1) ... Wool storage, sampling and testing industry (123)

Q13 How many people in total (i.e., full time, part time and casual) does your business currently employ?

________________________________________________________________

Q14 How many locations does the business operate from?

________________________________________________________________

Q15 How many business partners do you have that are active in the business?

________________________________________________________________

Q16 Would you describe your business as a family business?

- Yes (1)
- No (2)

Q17 How many years have you been a business owner?

________________________________________________________________

Q18 How many hours do you work in your business on average per week?

________________________________________________________________

Q19 Please give your best estimate of your revenue (e.g. sales turnover) for the last 12 months?

▼ $0 to less than $50k (1) ... $10m or more (6)

Q20  Impact of Covid19 on your business revenue (sales turnover)

|  | Large negative impact (1) | Some negative impact (2) | No impact (3) | Some positive impact (4) | Large positive impact (5) |
| --- | --- | --- | --- | --- | --- |
| 1 (1) |  |  |  |  |  |

Q21 On average, how often have you communicated with your accountant/bookkeeper/financial planner in the last 12 months? (i.e. the person who invited you to participate in this study)

▼ Never (1) ... Daily (8)

Q22 Do you have **face-to-face** meetings with your accountant/bookkeeper/financial planner?

- Yes (1)
- No (2)
- Sometimes (3)

Skip To: Q24 If Do you have face-to-face meetings with your accountant/bookkeeper/financial planner? = No

Skip To: Q23 If Do you have face-to-face meetings with your accountant/bookkeeper/financial planner? = Yes

Skip To: Q23 If Do you have face-to-face meetings with your accountant/bookkeeper/financial planner? = Sometimes

Q23 When did you last meet with them **face-to-face**?

________________________________________________________________

Q24 Please indicate your highest level of formal education

▼ Postgraduate Degree (1) ... Year 11 or below (includes Certificate 1/11) (7)

Q25 Your home postcode

________________________________________________________________

Q26  What do you feel is the level of your financial stress today?

|  | Overwhelming stress  1 (1) | 2 (2) | 3 (3) | 4 (4) | 5 (5) | 6 (6) | 7 (7) | 8 (8) | 9 (9) | No stress at all  10 (10) |
| --- | --- | --- | --- | --- | --- | --- | --- | --- | --- | --- |
| 1 (1) |  |  |  |  |  |  |  |  |  |  |

Q27 Using the steps below, indicate how satisfied you are with your present financial situation.

|  | Disatisified  1 (1) | 2 (2) | 3 (3) | 4 (4) | 5 (5) | 6 (6) | 7 (7) | 8 (8) | 9 (9) | Satisfied  10 (10) |
| --- | --- | --- | --- | --- | --- | --- | --- | --- | --- | --- |
| (1) |  |  |  |  |  |  |  |  |  |  |

Q28 How do you feel about your current financial situation?

|  | Feel overwhelmed  1 (1) | 2 (2) | 3 (3) | 4 (4) | 5 (5) | 6 (6) | 7 (7) | 8 (8) | 9 (9) | Feel Comfortable  10 (10) |
| --- | --- | --- | --- | --- | --- | --- | --- | --- | --- | --- |
| (1) |  |  |  |  |  |  |  |  |  |  |

Q29 How often do you worry about being able to meet monthly living expenses?

|  | Worry All the Time  1 (1) | 2 (2) | 3 (3) | 4 (4) | 5 (5) | 6 (6) | 7 (7) | 8 (8) | 9 (9) | Never Worry  10 (10) |
| --- | --- | --- | --- | --- | --- | --- | --- | --- | --- | --- |
| (1) |  |  |  |  |  |  |  |  |  |  |

Q30 How confident are you that you could find the money to pay for a financial emergency that costs about $1,000?

|  | No Confidence  1 (1) | 2 (2) | 3 (3) | 4 (4) | 5 (5) | 6 (6) | 7 (7) | 8 (8) | 9 (9) | High Confidence  10 (10) |
| --- | --- | --- | --- | --- | --- | --- | --- | --- | --- | --- |
| (1) |  |  |  |  |  |  |  |  |  |  |

Q31   How often does this happen to you? You want to go out to eat, go to a movie or do something else and don't go because you can't afford to?

|  | All the Time  1 (1) | 2 (2) | 3 (3) | 4 (4) | 5 (5) | 6 (6) | 7 (7) | 8 (8) | 9 (9) | Never  10 (10) |
| --- | --- | --- | --- | --- | --- | --- | --- | --- | --- | --- |
| (1) |  |  |  |  |  |  |  |  |  |  |

Q32 How frequently do you find yourself just getting by financially and living paycheck to paycheck?

|  | All the Time  1 (1) | 2 (11) | 3 (2) | 4 (3) | 5 (4) | 6 (5) | 7 (6) | 8 (7) | 9 (8) | Never  10 (9) |
| --- | --- | --- | --- | --- | --- | --- | --- | --- | --- | --- |
| (1) |  |  |  |  |  |  |  |  |  |  |

Q33 How stressed do you feel about your personal finances in general?

|  | Overwhelming Stress  1 (1) | 2 (2) | 3 (3) | 4 (4) | 5 (5) | 6 (6) | 7 (7) | 8 (8) | 9 (9) | No Stress at All  10 (10) |
| --- | --- | --- | --- | --- | --- | --- | --- | --- | --- | --- |
| (1) |  |  |  |  |  |  |  |  |  |  |

Q34  How well does the following describe your current money situation?

|  | Strongly disagree  1 (1) | Somewhat disagree  2 (2) | Neither agree or disagree  3 (3) | Somewhat agree  4 (4) | Strongly agree  5 (5) |
| --- | --- | --- | --- | --- | --- |
| Because of my money situation, I feel I will never have the things I want in life. (1) |  |  |  |  |  |
| I am behind with my finances. (2) |  |  |  |  |  |
| My finances control my life. (3) |  |  |  |  |  |
| Whenever I feel in control of my finances, something happens that sets me back. (4) |  |  |  |  |  |
| I am unable to enjoy life because I obsess too much about money. (5) |  |  |  |  |  |

Q35 Please rate the following statements regarding your business advisor (i.e. the accountant, bookkeeper or financial advisor who sent you the invitation).

|  | Strongly disagree (1) | Disagree (2) | Somewhat disagree (3) | Neither agree nor disagree (4) | Somewhat agree (5) | Agree (6) | Strongly agree (7) |
| --- | --- | --- | --- | --- | --- | --- | --- |
| I regularly seek out business advice from my business advisor. (1) |  |  |  |  |  |  |  |
| I can turn to my business advisor when things are going badly in the business. (2) |  |  |  |  |  |  |  |
| My business advisor knows when I'm doing it tough. (3) |  |  |  |  |  |  |  |
| My business advisor checks in with me to see how I'm doing. (4) |  |  |  |  |  |  |  |
| My business advisor provides advice on a range of business matters such as strategic planning, risk management and staffing matters. (5) |  |  |  |  |  |  |  |
| I use my business advisor for all my required financial administration. (6) |  |  |  |  |  |  |  |

Q36 In the past four weeks, how often did you feel….

|  | None of the time (1) | A little of the time (2) | Some of the time (3) | Most of the time (4) | All of the time (5) |
| --- | --- | --- | --- | --- | --- |
| Nervous (1) |  |  |  |  |  |
| Hopeless (2) |  |  |  |  |  |
| Restless (3) |  |  |  |  |  |
| That everything was an effort (4) |  |  |  |  |  |
| So depressed that nothing could cheer you up (5) |  |  |  |  |  |
| Worthless (6) |  |  |  |  |  |

Q36b How attentive of your mental health is your business advisor?

- Not attentive (we never talk about it) (1)
- Somewhat attentive (they politely enquire how I am, but we don’t explore it further) (2)
- Very attentive (they genuinely want to know how I am, and we talk about it) (3)

Q36c

|  | Never (1) | Once (2) | Occasionally (3) | Frequently (4) |
| --- | --- | --- | --- | --- |
| In the last month, did your business advisor talk to you about a mental health problem you may be experiencing? (4) |  |  |  |  |

Display This Question:

If Click to write the question text = Never

Q36d Why didn't you discuss your mental health with your business advisor? (select all that apply)

- Didn't have a problem to share (1)
- Didn't feel comfortable sharing it with them (2)
- Preferred to discuss it with someone else (3)
- They didn't ask (5)
- Other (4) ________________________________________________

Display This Question:

If Click to write the question text != Never

Q36c If you talked with your business advisor about your mental health problems over the past month, please indicate which of the following actions they have taken (select all that apply):

|  | Never (1) | Once (2) | A few times (3) | Many times (4) |
| --- | --- | --- | --- | --- |
| Spent time listening to my problems (1) |  |  |  |  |
| Helped calm me down (2) |  |  |  |  |
| Helped deal with the business-related issue contributing to the problem (3) |  |  |  |  |
| ​​​​​​​Enlisted others to help (4) |  |  |  |  |
| Encouraged self-help strategies (i.e. books, meditation, website) (5) |  |  |  |  |
| Recommended that I seek professional help (e.g. GP, crises support)   (7) |  |  |  |  |
| Talked to me about suicidal thoughts (8) |  |  |  |  |
| Anything else (9) |  |  |  |  |

Q37 How would you rate your levels of confidence and resilience?

|  | Strongly disagree (1) | Disagree (2) | Somewhat disagree (3) | Somewhat agree (4) | Agree (5) | Strongly agree (6) |
| --- | --- | --- | --- | --- | --- | --- |
| I feel confident in representing my company in meetings with prospective clients. (1) |  |  |  |  |  |  |
| I feel confident actioning the company's strategy. (2) |  |  |  |  |  |  |
| I feel confident presenting information to a group of colleagues. (3) |  |  |  |  |  |  |
| I usually take stressful things at work in my stride. (4) |  |  |  |  |  |  |
| I can get through difficult times at work because I've experienced difficulty before. (5) |  |  |  |  |  |  |
| I can be "on my own" so to speak to work if I have to. (6) |  |  |  |  |  |  |

Q38 How easy is it to talk to the following people?

|  | 1  Very difficult (1) | 2 (2) | 3 (3) | 4 (4) | 5  Very easy (5) |
| --- | --- | --- | --- | --- | --- |
| Clients (1) |  |  |  |  |  |
| Family and friends (2) |  |  |  |  |  |
| Colleagues and peers (3) |  |  |  |  |  |

Q39 To what extent are the following people willing to listen to your problems?

|  | 1  Not at all (1) | 2 (2) | 3 (3) | 4 (4) | 5  A great deal (5) |
| --- | --- | --- | --- | --- | --- |
| Clients (1) |  |  |  |  |  |
| Family and friends (2) |  |  |  |  |  |
| Colleagues and peers (3) |  |  |  |  |  |

Q40 In general, would you say your health is:

|  | Excellent (1) | Very good (2) | Good (3) | Fair (4) | Poor (5) |
| --- | --- | --- | --- | --- | --- |
| (6) |  |  |  |  |  |

Q41 The following two questions are about activities you might do during a typical day. Does **YOUR HEALTH NOW LIMIT YOU** in these activities? If so, how much?

|  | Yes, Limited A Lot (1) | Yes, Limited A Little (2) | No, Not Limited At All (3) |
| --- | --- | --- | --- |
| MODERATE ACTIVITIES, such as moving a table, pushing a vacuum cleaner, bowling, or playing golf: (1) |  |  |  |
| Climbing SEVERAL flights of stairs: (2) |  |  |  |

Q42 During the **PAST 4 WEEKS** have you had any of the following problems with your work or other regular activities **AS A RESULT OF YOUR PHYSICAL HEALTH?**

|  | Yes (1) | No (2) |
| --- | --- | --- |
| **ACCOMPLISHED LESS** than you would like: (1) |  |  |
| Were limited in the **KIND** of work or other activities: (2) |  |  |

Q43 During the **PAST 4 WEEKS**, were you limited in the kind of work you do or other regular activities **AS A RESULT OF ANY EMOTIONAL PROBLEMS** (such as feeling depressed or anxious)?

|  | Yes (1) | No (2) |
| --- | --- | --- |
| **ACCOMPLISHED LESS** than you would like: (1) |  |  |
| Didn’t do work or other activities as **CAREFULLY** as usual: (2) |  |  |

Q44 During the **PAST 4 WEEKS**, how much did **PAIN** interfere with your normal work (including both work outside the home and housework)?

|  | Not at all (1) | A little bit (2) | Moderately (3) | Quite a bit (4) | Extremely (5) |
| --- | --- | --- | --- | --- | --- |
| (1) |  |  |  |  |  |

Q45 The next three questions are about how you feel and how things have been **DURING THE PAST 4 WEEKS**. For each question, please give the one answer that comes closest to the way you have been feeling. How much of the time during the **PAST 4 WEEKS** –

|  | All of the Time (1) | Most of the Time (2) | A good Bit of the Time (3) | Some of the Time (4) | A Little of the Time (5) | None of the Time (6) |
| --- | --- | --- | --- | --- | --- | --- |
| Have you felt calm and peaceful? (1) |  |  |  |  |  |  |
| Did you have a lot of energy? (2) |  |  |  |  |  |  |
| Have you felt downhearted and blue? (3) |  |  |  |  |  |  |

Q46 During the **PAST 4 WEEKS**, how much of the time has your **PHYSICAL HEALTH OR EMOTIONAL PROBLEMS** interfered with your social activities (like visiting with friends, relatives, etc.)?

|  | All of the time (1) | Most of the Time (2) | A Good Bit of the Time (3) | Some of the Time (4) | A Little of the Time (5) | None of the Time (6) |
| --- | --- | --- | --- | --- | --- | --- |
| (1) |  |  |  |  |  |  |

Q47 To what extent do you agree with the following statements regarding your business advisor (accountant, bookkeeper, financial planner)

|  | Strongly disagree (1) | Disagree (2) | Neither agree or disagree (3) | Agree (4) | Strongly agree (5) |
| --- | --- | --- | --- | --- | --- |
| My business advisor never withholds critical information that might affect my decision-making (2) |  |  |  |  |  |
| I am confident in my business advisor’s ability to provide effective general business advice (1) |  |  |  |  |  |
| My business advisor helps me improve the performance of my business (9) |  |  |  |  |  |
| My business advisor can be trusted at all times (6) |  |  |  |  |  |
| My business advisor is always honest & truthful (4) |  |  |  |  |  |
| My business advisor can be regarded as credible (11) |  |  |  |  |  |
| My business advisor is sincere in their dealings with my business (12) |  |  |  |  |  |
| My business advisor always acts in the best interests of my business (7) |  |  |  |  |  |
| My business advisor takes care of my needs as a client (5) |  |  |  |  |  |
| My business advisor has a high level of integrity (10) |  |  |  |  |  |
| My business advisor puts my business’s interests above their own (8) |  |  |  |  |  |

# Interview for Business Advisors

|  | Background | Outcome |
| --- | --- | --- |
| 1 | Can you describe your role and what you do?  What types of advice do you provide to your SME clients? | Warm up questions |
| 2 | How often do you typically meet with your SME clients (including any that you invited to take part)?  Is this face to face, zoom, email?  How long is your typical relationship? | Warm up questions |
| 3 | How have your clients coped during the pandemic? | Background, context |
| 4 | Have you noticed a change in their mental health during this time? Can you describe an example of this? | Background, context |
| 5 | Do you think it’s part of your role to support the mental health of your SME clients? | Background, context |
| 6 | And, how have you been coping lately? Have you found it stressful or were you able to cope?  Has this affected your wellbeing or mood? | BA wellbeing |
|  | Relationship Building Training | |
| 7 | What did you learn from the Relationship Building Training? | Efficacy  Maintenance  RBT- for those who did it. |
| 8 | How useful did you find the Relationship Building Training on a scale of 1 to 10 (1 not useful, 10 very useful).  Why?  Which topic, if any, did you find not particularly useful? | Utility |
| 9 | How have you used what you learnt from Relationship Building Training? Can you give me any examples?  Examples  Do you show more an interest in what is important to your small-medium business clients?  Do you listen more to what they’re saying?  Do you gain greater insight into SME’s business? And in turn, are you able to provide higher quality advice?  Do you make more of an effort to work together with SME to find solutions to their financial problems?  If you haven’t used any of it, why not? | Efficacy  Maintenance  Delivery RBT |
| 10 | How confident do you feel using the Relationship Building Skills you learnt from the training?  Or were you always confident in your skills to build more trusting relationships and communicate with clients? |  |
|  | Mental Health First Aid | |
| 11 | What did you learn from the Mental Health First Aid training? |  |
| 12 | How useful did you find the MHFA on a scale of 1 to 10 (1 not useful, 10 very useful).  Why?  Was there any topic you didn’t find useful? |  |
| 13 | How have you used what you learnt from the MHFA training? For example, have you used ALGEE? Can you give me any examples?  What types of advice did you provide? i.e refer them to their GP/beyond blue/lifeline? | Provision of MHFA |
| 14 | (If yes to above), what encouraged you to have a conversation about their mental health?  Examples of enabling factors: keen to practice, felt they were needed, feel a moral obligation, know the SMEs don’t have a strong support system. |  |
| 15 | What about just checking in, do you ever get in touch with your clients to see how they’re going? | Quality of the relationship/Provision of MHFA |
| 16 | Do you do this more, less or the same amount since you completed the training? |  |
| 17 | What sort of things, if any, make you feel hesitant about having conversation with your clients about their mental health?  e.g. time, professional boundaries, SMEs unwilling to share information. | Barriers |
| 18 | (If no to earlier MFHA question) Why haven’t you used it?  Haven’t needed to? Or forgot the skills, no confidence to use them, no time. |  |
| 19 | Has your confidence to have personal conversations with your clients changed since you did the training?  Or is this something you were confident doing before the training? | Confidence to deliver MHFA. |
|  | General Impact | |
| 20 | Has the training changed the strength of the relationship you have with your clients?  OR  Do you feel you have a stronger bond with your SME clients because of the training?  For example:  Do you feel you have better insight into the SME’s business and/or how they’re coping?  Are you able to provide more useful advice? | Quality of the relationship.  Note: quality of relationship and quality of advice do not always coincide.  Could also be enhancing intimacy, part of trust. |
| 21 | Since completing the training, do you feel like you better understand when your clients are doing it tough?   (i.e. listen more, ask more questions) | Quality of the relationship. |
| 22 | Do you think your clients have more success with their business because of your help?  Have you helped your client’s business grow in ways that they could not have done on their own?  If yes, has the program helped you with this in any way?  For example:   - You are better able to guide clients through different processes (i.e. making claims) - Clients feel they are receiving advice not just told “yes” or “no” - You point out things over and above the client’s expectations   If yes, would you say the RBT or MHFA or both has had the biggest impact? | Quality of business advice  Trust: Competence |
| 23 | Has the training helped you to better alleviate your client’s financial distress? How? (i.e. better listener, worked together to find solutions)  Can you think of an example of how you helped a SME client?  Note: financial distress means feeling your finances are out of control and feeling financially insecure because you may have cash flow and/or sale issues. | Quality of business advice  Relationship between BA advice and SME financial wellbeing. |
| 24 | (if yes to above) Do you think alleviating your client’s financial distress also improved their state of mind/wellbeing/mood?  Do you have any examples you’d like to share? | Relationship between SME financial distress and SME overall wellbeing |
| 25 | On a scale of 1 to 10, to what extent do you feel like you and your SME clients are on the same page about their business needs?  With 1 being not on the same page, and 10 being 100% on the same page.  Why?  Has this changed since you took part in the training? | Trust: Information asymmetry |
| 26 | How has the training affected your own wellbeing/mental health/mood?  i.e. are you more aware of your own wellbeing/mental health/mood? | Efficacy  Quality of life/psychological distress |
|  | Closing questions |  |
| 27 | Why did you decide to participate in Counting on U?  i.e. was there a time when a client revealed that they were struggling financially and/or emotionally and you didn’t know how to respond? | Reach  Adoption |
| 28 | How useful did you find the Booster session? |  |
| 29 | Do you know of any colleagues who decided not to participate in the program? Do you know their reasons why? |  |
| 30 | Have there been any negative outcomes from participating in CoU? | Unintended consequences |
| 31 | Is there anything we could do to improve the program? |  |
| 32 | How likely are you to recommend CoU to your friends or colleagues on a scale of 1 to 10. 1 being not likely and 10 will recommend. |  |
| 33 | Is there anything else you would like to add? (about any topic) | Other |

# SME Interview

|  | Question | Outcome measured |  |
| --- | --- | --- | --- |
| 1 | Can you tell me a bit about your business?  How long have you owned your business?  Is this the first time you’ve owned a business?  How many employees do you have?  Where are you based? | Background |  |
| These next few questions are about your experiences as a business owner, challenges you face in this role and your experiences over the last 12 months. (personal costs and stressors) | | | |
| 2 | The last couple of years has been very challenging for many businesses. How has your business been going lately?  Clarify: So your business is going through a period of growth OR the business is pretty stable at the moment OR your business has been impacted quite a bit by the pandemic? | Background  Determinants of SMEs need for advice |  |
| 3 | And how have you personally found the past couple of years to be as a business owner? For example, did you find it quite stressful or easy to manage? | SME wellbeing |  |
| 4 | How do you feel about your business’s financial situation at the moment? Why?  Clarify: So it sound as though you’re feeling a bit stressed about the business at the moment OR it sounds like you feel ok with how your business is going at the moment OR it sounds like you feel pretty confident about how your business is going at the moment  What aspects of owning a business do you find most challenging or stressful?  Has this changed since six months ago? | SME wellbeing: financial |  |
| 5 | What aspects of owning a business do you find the most challenging?  Has this changed from six months ago? | Determinants of SME wellbeing/stressors |  |
| 6 | And what about your current wellbeing/mood?  Has this changed from six months ago? | SME wellbeing |  |
| 7 | There is a known link between financial stress and wellbeing. Is this something that you see as relating to your own experience? |  |  |
| 8 | Does owning a business impact your overall wellbeing or ability to enjoy life? If so, in what way? | SME wellbeing |  |
| The following questions are about the business advisor who invited you to take part and your experiences working with them. | | | |
| 9 | The person who invited you to participate in the study, what is their role e.g. accountant/bookkeeper/financial planner?  When did you last meet with your business advisor?  Was this face-to-face?  How often have you met up over the last 6 months? |  |  |
| 10 | How satisfied are you with the advice or services they provide you?  Do you feel they provide good quality advice?  Do you feel they put in effort, or work hard for you?  Has this changed from six months ago? | Quality of BA Advice  Trust: Competence |  |
| 11 | What types of services or advice do they provide you?  Does your accountant/bookkeeper provide basic services only or do they also provide business advice?  Note:  Basic (traditional) services: taxation, compliance services, audit services  Other (non-traditional) services: financial services (cash flow, banking, super, asset structuring), business operational services (lease negotiation, payroll, HR, IT), business dynamics (purchase, transfer or sale of business), intangible services (sounding board, friend, psychologist), family services (wills, estate planning, succession planning)  Are there any other services you would like from your business advisor? | BA: Background  Trust: Competence |  |
| 12 | So for the next couple of questions, we are interested in finding out whether your accountant/bookkeeper has had an impact on your financial and general wellbeing. So to start with…  Has your accountant/bookkeeper had an impact on your financial wellbeing? (this could be positive or negative) If yes, what type of impact have they had?  Note: financial wellbeing is feeling in control of your finances and feel financially secure, now and in the future.  Has this changed from six months ago? | Relationship between BA advice and SME financial wellbeing |  |
| 13 | (continuation from above) Has your business advisor had an impact on your overall wellbeing? For example, this could be your physical wellbeing or your mood or mental health?  Has this changed from six months ago? | Relationship between BA advice and SME overall wellbeing |  |
| The next questions are about the quality of the relationship between you and your business advisor. | | |  |
| 14 | How close would you describe your relationship with your BA?  Does your BA ever share personal details about their life with you?  Can you think of any scenarios when they have demonstrated this?  Has this changed from six months ago? | Trust: Enhance intimacy |  |
| 15 | Do you turn to your BA when faced with challenges in your business? Why, why not?  Have you needed to do this over the last 6 months?  Can you explain what happened?  Has this changed from six months ago? | Quality of relationship |  |
| 16 | Is there anyone else you turn to for support when faced with challenges in your business? |  |  |
| 17 | Do you turn to your BA for support with your wellbeing? E.g. when you may be struggling personally. Why, why not?  Have you needed to do this over the last 6 months?  Can you explain what happened?  Has this changed from six months ago? |  |  |
| 18 | Is there anyone else you turn to for support with your wellbeing? |  |  |
| 19 | Does your BA check in to see how you’re doing?  How have they done this?  Has the extent to which they check in increased, decreased or stayed the same compared with 6 months ago?  If yes,  Do you believe they more sincere when they check in with you?  Do you feel they are listening more? | Quality of the Relationship/Provision of MHFA |  |
| 20 | Over the last 6 months, has your BA had a conversation with you about your wellbeing? i.e., any personal struggles you may be experiencing with your family or relationships, or to do with stress, anxiety, depression etc.  If yes, would you feel comfortable describing that situation, in as little or as much detail as you would like?  Did your BA make any suggestions on what you could do to help with that situation?   - self-help strategies (i.e., exercise)? OR - services such (your GP, Beyondblue, lifeline, financial counselling) | Provision of MHFA |  |
| 21 | If no to above, why is that? E.g.   - you haven’t had any personal struggles - you’ve turned to someone else for advice - you didn’t feel comfortable sharing them with your BA? | Provision of MFHA |  |
| 22 | If yes to above, how helpful was your BA in this situation?  Was there anything they said that was particularly helpful or unhelpful?  Did you feel they were being non-judgemental? | Provision of MHFA |  |
|  | Have you had conversations about your wellbeing like this, more or less or the same compared with 6 months ago? |  |  |
| 23 | To what extent do you feel like you and your accountant/bookkeeper are on the same page about your business needs?  Do you feel like your accountant/bookkeeper ensures you fully understand their advice? | Trust: Information asymmetry |  |
| 24 | Does your BA listen carefully/thoughtfully when you are speaking or when you raise concerns? I.e., are they attentive?  Do they enquire more deeply when you raise business concerns?  Are they empathetic when discussing problems?  Has this changed since we last met? | Trust: Empathy |  |
| 25 | Do they work collaboratively with you when solving problems?  Can you think of any scenarios when they have demonstrated this?  Has this changed since we last met? | RBT training communication skills: work collaboratively |  |
| 26 | Do think your BA shares all of the critical information that might affect your decision making?  Do you feel like your accountant/bookkeeper ensures you fully understand their advice?  Do you feel comfortable sharing information with your BA?  Has this changed from 6 months ago? | Trust: Information asymmetry |  |
| 27 | Do you trust your BA?  How open and honest do you believe your BA to be?  Do they admit any short comings in their knowledge?  Has this changed from 6 months ago? | Trust: Integrity |  |
| The next couple of questions are about your experiences participating in Counting on U. | | | |
| 28 | Why did you decide to participate in Counting on U?  Was there anything that made it difficult for you to participate in Counting on U?  Was there anything that helped or encouraged you to participate in Counting on U? | Reach |  |
| 29 | Is there anything we could have done to make your participation easier? | Reach |  |
| 30 | Is there anything else you would like to add? (about any topic) | Other |  |

# Trainer Interview Guide

| N | Questions | Outcome |
| --- | --- | --- |
| 1 | Why did you decide to become a Counting on U trainer? | Warm up |
| 2 | What did you like about the Counting on U program? |  |
| 3 | What did you not enjoy about the program? | Implementation (improvements) |
| Relationship Building Training (RBT)  These next few questions are going to be specifically about the RBT session. | | |
| 4 | What was your experience of delivering the RBT?  Possible prompts:  What were the best/worst parts?  Were there any challenges you faced? | Implementation (changes to the programme) |
| 5 | How useful do you think the RBT session is? | Efficacy (understanding course content) |
| 6 | Do you think the participants valued learning RBT?  Was there any content the participants really valued in particular?  Optional extension questions:  How engaged were the participants in the sessions?  Was there any content that the participants struggled with?  Conversely, | Efficacy (understanding course content)  Implementation (engagement of participants) |
| 7 | Do you have any suggestions on how we could improve the RBT session?  Optional extension question:  Were the case studies used in the videos appropriate? | Implementation (changes to the programme) |
| Booster sessions | | |
| 8 | What was your experience of delivering the Booster sessions? | Implementation (changes to the programme) |
| 9 | How useful do you think the Booster sessions are? | Implementation (engagement of participants) |
| 10 | Do you think the participants appreciated the value of having Booster sessions?  Was there any content the participants really valued in particular?  *Optional extension question:*  *How engaged were the participants in the sessions?*  *Was there any content the participants struggled with?* | Efficacy (understanding course content)  Implementation (engagement of participants) |
| 11 | Do you have any suggestions on how we could improve the Booster sessions? | Implementation (changes to the programme) |
| MHFA sessions | | |
| 12 | What was your experience of delivering the MHFA sessions? | Implementation (changes to the programme)  Note: MHFA was adapted to suit the BA audience |
| 13 | Was this different when delivering MHFA vs. RBT and Booster sessions? | Implementation (changes to the programme) |
| 14 | Do you think the participants appreciated the importance of learning MHFA?  Was there any content the participants really valued in particular?  Optional extension questions:  How engaged were the participants in the sessions?  Was there any content that the participants struggled with? | Efficacy (understanding course content)  Implementation (engagement of participants) |
| Learning the course content | | |
| 15 | Can you describe and compare a typical participant who was more engaged and less engaged in the program? | Reach (motivations behind participation) |
| 16 | Can you recall any stories or anecdotes from the accountants/bookkeepers who had used what they learnt?  Note: deidentifying any names. | Efficacy (understanding course content) /Implementation (engagement of participants) |
| Experience as a trainer delivering a remote research training program | | |
| 17 | What were some of the challenges you faced when providing training? | Implementation (improvements) |
| 18 | What was your experience like delivering zoom-based sessions?  What impact, if any, do you think having zoom sessions had on attendance and engagement in the sessions (compared with face-to-face)?  Did you feel the training provided a safe environment for participants? | Implementation (improvements)  Reach (motivations behind participation) |
| 19 | Had you been provided with all the resources you need to deliver the training? If no, what other resources did you need? | Implementation (resources) |
| 20 | Do you have any recommendations on how we can Improve Counting on U? | Implementation (improvements) |
| 21 | Did you change any aspects of the training from what was intended? If yes, what and why? | Implementation (fidelity) |
| 22 | Have there been any negative outcomes that you personally or professionally experienced from being a Counting on U trainer? | Implementation (unintended consequences) |
| 23 | Have any new opportunities come about because you took part in Counting on U? | Implementation (unintended consequences) |
| 24 | What has been the experience like working with MWW and other trainers when delivering Counting on U? | Implementation (resources) |
| 25 | Is there any way we can improve how you work with MWW or Deakin? | Implementation (resources) |
| Insights into participant’s experience | | |
| 27 | What do you think some of the reasons are that participants attended training?  What do you think some of the reasons are that participants did not attend training?  Is there anything we could do to improve participant attendance? | Reach (motivations behind participation) |
| 28 | Is there any other feedback you received from participants that you would like to share?  Optional extension questions:  Did you experience any challenges getting participants to complete the surveys?  Do you have any suggestions on how we can increase the survey response rate? | Reach (motivations behind participation) |
| 29 | Can you think of any negative outcomes’ participants may have experienced from taking part in the training? | Implementation (unintended consequences) |
| Summary questions | | |
| 30 | What are your thoughts on the value of CoU? |  |
| 31 | Is there anything else you would like to add? (about any topic) |  |

# Training Vendor Interview Guide

|  | *General introduction* |  |
| --- | --- | --- |
| 1 | Can you tell me a little about your organisation?  What does your organisation do? | Type of organisation who adopted the program [ADOPTION] |
| 2 | What has been your role in Counting on U? | General introduction |
|  | *Exploring the value of Counting on U* |  |
| 3 | Why did your [organisation] decide to get involved in Counting on U? | Level of support for program [ADOPTION]  Remove when interviewing MWW/HumanEx |
| 4 | How would you describe what Counting on U is hoping to achieve? | Level of support for program [ADOPTION] |
| 5 | How valuable or useful do you believe Counting on U to be? | Level of support for program [ADOPTION] |
| 6 | We want to find out a bit about your perspectives on participants experiences taking part in the program.  What types of feedback are you receiving?  Positive or negative  Did participants appear to value the program  What is your understanding of why people are signing up to take part. | Level of support for program [ADOPTION] |
| 7 | Are you able to share anything about the trainer’s experiences taking part in Counting on U?  Feeback you have received  Positive or negative  Did they value the program? | [Level of support for program [ADOPTION] |
|  | *Review the roll-out of Counting on U* |  |
| 8 | What things about CoU have worked best?  What has helped to keep CoU running? | Facilitators to participating [ADOPTION] |
| 9 | What has been some of the challenging aspects of working on Counting on U?  What hasn’t worked?  Including:  Working with Deakin  Working with member bodies/other partners or organisations  Dealing with participants | Barriers to participating [ADOPTION]  We can remove negative outcome question below |
| 10 | What resources has your organisation needed to implement CoU?  i.e, staffing, money, systems, tech  Was it enough? | Costs/resources needed [IMPLEMENTATION] |
| 11 | Did you feel like you had the support you needed from others within your organisation to work on the program? | Level of support from peak bodies [ADOPTION] |
| 12 | Did you feel like you had the support you needed from Deakin to deliver the program? | Facilitators/barriers to participating [ADOPTION] |
| 13 | How did the roll out of this program compare to other training programs your organisation has been involved in? | Costs/resources needed [IMPLEMENTATION] |
| 14 | What have been the key learnings from when we started the program until now?   - What were some of the positive changes we made? - What were some of the negative changes we made? | Changes to the program [IMPLEMENTATION] |
| 15 | Would you like to comment on any of the following aspects of the training?  Managing participant’s emails  Managing the booking/scheduling  Managing the trainers  Managing the delivery of the training sessions  Online systems/platforms | Facilitators/barriers to participating [ADOPTION] |
| 16 | Have there been any unintended consequences because of your organisation taking part in CoU?  To:  -Yourself  -Your organisation  -Your members | Unintended consequences [IMPLEMENTATION~~]~~ |
|  | *We are now going to discuss your thoughts on the future of Counting on U*  Follow-up interview may be needed to ask about future of CoU/how sustainable/their support? (i.e., cut backs/whether they can deliver it themselves/is it a luxury)?. |  |
| 17 | Is there any way we can improve Counting on U?  **Or**  Is there anything that you would suggest changing in the future? | Changes to the program [IMPLEMENTATION] |
| 18 | Is your organisation interested in continuing to deliver Counting on U in the future?  Why/why not? | Institutional sustainability of the program [MAINTENANCE] |
| 19 | What is your organisation’s preferred format to continue to deliver the program? | Institutional sustainability of the program [MAINTENANCE] |
| 20 | What challenges could affect your organisations continued delivery of Counting on U in the future?  How sustainable is it?, i.e., resources? Time? Money? | Institutional sustainability of the program [MAINTENANCE] |
| 21 | Is there any way Deakin can help to support the continued delivery of Counting on U in the future? | Institutional sustainability of the program [MAINTENANCE] |
| 22 | Do you have any further comments you would like to add about anything we have discussed today or anything else? |  |

# Partner Interview Guide

|  | *General introduction* |  |
| --- | --- | --- |
| 1 | Can you tell me a little about your organisation?  How many members?  What does your organisation do for your members? | Type of organisation who adopted the program [ADOPTION] |
| 2 | What has been your role in Counting on U? | General introduction |
|  | *Exploring the value of Counting on U* |  |
| 3 | Do you know why your [organisation] decided to get involved in Counting on U? | Level of support for program [ADOPTION]  Remove when interviewing MWW/HumanEx |
| 4 | How would you describe what Counting on U is hoping to achieve? | Level of support for program [ADOPTION] |
| 5 | How valuable or useful do you believe Counting on U to be? | Level of support for program [ADOPTION] |
| 6 | Are you able to share anything about your members experiences taking part in Counting on U?  Positive or negative  Did they value the program | Level of support for program [ADOPTION] |
| 7 | For your members who completed training, why do you believe they decided to take part in Counting on U? | Recruitment facilitators  [REACH} |
| 8 | For your members who did not enrol what do you think are some of the reasons they decided to not take part? | Recruitment barriers  [REACH] |
| 9 | Is there anything we may have done to encourage more of your members to participate? | [REACH]. |
|  | *Review the roll-out of Counting on U* |  |
| 10 | What factors have worked or helped keep CoU running?  What’s worked | Facilitators to participating [ADOPTION] |
| 11 | What did your organisation do to do to help promote Counting on U?  i.e., did they go over and above to promote CoU? Video interviews? | Level of support from peak bodies [ADOPTION] |
| 12 | Was there a champion at your organisation who was invested in the success of this program? Including yourself?  What did they (or you) do? | Level of support from peak bodies [ADOPTION] |
| 13 | What has been some of the challenging aspects of working on Counting on U?  What hasn’t worked?  Including:  Working with Deakin  Other accounting/bookkeeper organisations  Dealing with members | Barriers to participating [ADOPTION]  We can remove negative outcome question below |
| 14 | What resources has your organisation needed to implement CoU?  i.e, staffing, money  Was it enough? | Costs/resources needed [IMPLEMENTATION] |
| 15 | How did the roll out of this program compare to other training programs your organisation has offered? | Costs/resources needed [IMPLEMENTATION] |
| 16 | Did you feel like you had the support you needed from your organisation to work on the program? | Level of support from peak bodies [ADOPTION] |
| 17 | Did you feel like you had the support you needed from Deakin to deliver the program? | Facilitators/barriers to participating [ADOPTION] |
| 18 | Would you like to comment on any of the following aspects of the training?  -Timeframes for member recruitment  -Scheduling of training session dates/times  -Eligibility criteria  -Marketing and communications  -CPD  -Online systems/platforms | Facilitators/barriers to participating [ADOPTION] |
| 19 | What have been the key learnings from when we started the program until now?   - What were some of the positive changes we made? - What were some of the negative changes we made? | Changes to the program [IMPLEMENTATION] |
| 20 | Have there been any unintended consequences because of your organisation taking part in CoU?  To:  -Yourself  -Your organisation  -Your members | Unintended consequences [IMPLEMENTATION~~]~~ |
|  | *We are now going to discuss your thoughts on the future of Counting on U*  Follow-up interview may be needed to ask about future of CoU/how sustainable/their support? (i.e., cut backs/whether they can deliver it themselves/is it a luxury)?. |  |
| 21 | Is there any way we can improve Counting on U?  **Or**  Is there any way we can improve your members’ experiences taking part Counting on U? | Changes to the program [IMPLEMENTATION] |
| 22 | Is your organisation interested in continuing to deliver Counting on U in the future?  Why/why not? | Institutional sustainability of the program [MAINTENANCE] |
| 23 | What is your organisation’s preferred format to continue to deliver the program? | Institutional sustainability of the program [MAINTENANCE] |
| 24 | What challenges could affect your organisations continued delivery of Counting on U in the future?  How sustainable is it?, i.e., resources? Time? Money? | Institutional sustainability of the program [MAINTENANCE] |
| 25 | Is there any way Deakin can help to support the continued delivery of Counting on U in the future? | Institutional sustainability of the program [MAINTENANCE] |
| 26 | Do you have any further comments you would like to add about anything we have discussed today or anything else? |  |
