# Supplementary material for: Counting on U training to enhance trusting relationships and mental health literacy among business advisors: protocol for a randomised controlled trial
Source: BMC Psychiatry. 2022 Jun 15;22:400. doi: 10.1186/s12888-022-04034-7 (PMC9199223; doi:10.1186/s12888-022-04034-7)
Supplement: Supplementary file 2 — Additional file 2. Plain Language Statement for business advisors. [file 12888_2022_4034_MOESM2_ESM.docx]

**Plain Language Statement**

**Full Project Title:** Counting On U: Supporting SME advisors and owners towards better mental health

**Principal Researcher(s):** Professor Andrew Noblet, Professor George Tanewski,

**Associate Researcher(s):** Ms Carol Leow-Taylor, Dr Leanne Saxon, Dr Sophie Bromfield, Dr Claudia Escobar Vega, Prof Michael Berk, Prof Arlene Walker, Prof Tony LaMontagne

For questions or concerns, please email the Deakin research team at: counting-on- [u@deakin.edu.au](mailto:u@deakin.edu.au)

Thank you for considering our invitation to take part in this research. Please read the following sections to understand what is required from you and where you can seek further information if required. Please know that your participation is completely voluntary, and you can withdraw from the project at any time. If you do decide to participate, we thank you for your contribution.

# What is Counting On U?

Counting On U is a 3-year intervention study being conducted across Australia which aims to provide training to business advisors to better support the mental health needs of small and medium enterprise owners. Mental illness is the single biggest cause of disability in Australia and small business owners are a large and vulnerable population that are often difficult to reach through traditional health intervention approaches. The Covid-19 pandemic has made the provision of support to this group particularly important as many small enterprise owners may be experiencing a heightened state of distress. In response to these needs, it has been identified that business advisors (e.g., auditors, accountants, tax advisors, financial planners, insolvency experts and other business finance professionals) are in a unique position to provide support to small enterprise owners due to their regular contact.

Mentally Well Workplaces, and our supporting partners Mental Health First Aid Australia will therefore be providing business advisors with training to improve their understanding of mental health and financial wellbeing and support them in the identification,

management and prevention of mental health conditions within the context of a business advisor–small business owner relationship. The training program will be conducted across 2021 and 2022 with follow up evaluation in 2023 to determine its effectiveness. By taking part in the program, you will receive professional development credits that fulfil part of your professional membership requirements.

# Who is conducting the project?

The project is being conducted by Deakin University, with the support of our partners IPA, CPA Australia, CAANZ, ICB, ICF, FPA, AFA, Mental Health First Aid Australia, Beyond Blue, Worksafe, and the Australian Treasury.

# Has this project been approved? Will it be monitored?

This project has been approved by the Deakin University Human Research Ethics Committee (HREC), the National Health and Medical Research Council of Australia (NHMRC), and the Australian Treasury. The Deakin research team will monitor the project’s progress and will report to Deakin University’s HREC.

# Who is funding this trial?

This study has received funding from the National Health and Medical Research Council of Australia (NHMRC), the Australian Treasury, and our partner organisations: Mental Health First Aid Australia, Beyond Blue, Institute of Public Accountants, and Worksafe Victoria.

# What does the project involve?

*Training*

All participants will complete Mental Health First Aid (MHFA) training. Participants will learn the following:

- the signs and symptoms of common and disabling mental health problems in adults;
- how to provide initial help;
- where and how to get professional help;
- what sort of help has been shown by research to be effective, and;
- how to provide mental health first aid in a crisis situation using a practical, evidence-based action plan.

The MHFA training will take approximately 10 hours in total to complete, comprising the following components:

- 5 hours of online e-learning completed in your own time, and;
- Two x 2.5 hours of online Zoom training sessions led by a Mentally Well Workplaces facilitator.

Half of the participants will be randomly selected to also undertake relationship building training (RBT). In this training, participants will learn about communication and rapport building, within the context of business advisor-small enterprise owner relationships.

This training will involve a:

- 2 hour online Zoom training session led by a Mentally Well Workplaces facilitator.

All participants will complete a brief, online booster session 1 and 3 months after completing the initial training. The booster session will take 60 minutes to complete and will be conducted online via Zoom.

*Surveys*

Several surveys will require completion as follows:

- Prior to participating in the training, your understanding and knowledge of mental health, relationships with your small enterprise owner clients, and your workrelated experiences will be assessed by completing a survey. You will also complete this survey during the 1-month booster session and 6 months after your initial training. These surveys also will take approximately 15 minutes to complete.
- A brief training feedback survey after the completion of each training session (approximately 5 minutes).

*Interviews*

A small number of participants (approximately 40) will be asked to take part in an in- depth interview regarding motivations for completing the training and the effectiveness of the program. Therefore, there is a possibility that you will be invited to participate in an interview after completing the training program.

*Recruitment of small and medium enterprise owners (Optional)*

As part of this project, we request that you assist in the nomination and hence recruitment of 1 or 2 small and medium enterprise owners to help evaluate the effectiveness of the training. These owners must:

- be an owner-manager who is one of your clients; and
- employ between 1 and 199 employees

Following completion of the registration process, you will be asked to email one to two small-to-medium enterprise (SME) owner clients with an invitation to participate. SME clients who agree to participate will be asked to complete three short surveys, and will be compensated with a $25 e-gift voucher for each survey completed. To maintain confidentiality of the small enterprise owner, owner/s who have consented to participate in the project will remain anonymous to you

# Will there be any reimbursement or incentives to participate?

You will not be paid for your participation in this project. However, as an incentive to participate, you will attain certification as an accredited mental health first aider. If you are a member of CPA, CA ANZ, ICB, IPA, or FPA, you will also receive between 12 to a maximum of 15 CPD points to count towards your structured learning professional development requirements.

Upon completion of all three surveys, you will be entered into a prize draw to win a

**$6000 travel voucher**. The prize will be drawn in October 2023 and will be valid for a period of three years. You must be an Australian resident to be eligible to win the prize. For terms and conditions please [click here](https://blogs.deakin.edu.au/counting-on-u/wp-content/uploads/sites/392/2021/08/Competition-Terms-and-Conditions_CoUCompetition.pdf).

As an incentive, we will also offer you a **$30 gift voucher** for the completion of Survey 2 & 3.

# Booking policy for training activities

As a courtesy to fellow business advisers, please contact us as soon as you know that you are no longer able to attend training on your allocated date/time so we can source an alternate date for you. You can re-schedule your sessions up to two times, depending on availability of alternate session dates/times:

- Re-scheduling an RBT session may (or may not) result in a re-scheduling of your two MHFA sessions
- Re-scheduling the first MHFA session will result in a re-scheduling of both MHFA sessions

If, due to extenuating circumstances, you are unable to attend RBT training on the day, please contact us and we will organise for you to watch a recording of the Zoom session. The recording will need to be viewed in order for you to receive certification and CPD points for Counting on U. No recordings are available for MHFA sessions due to the content of topics being discussed

# Why were you chosen for this research?

You were chosen for this research as you provide advice to small and medium enterprise owners. Therefore, you are in a unique position to help provide support to this vulnerable and often difficult to reach population.

# Consenting to participate in the project and withdrawing from the research

Consent to participate in this research is obtained from you, the participant, through completion of the consent form. You are able to withdraw from the research at any stage by contacting your representative at the relevant email address below listed below:

- - CAANZ members, please email: [cawellbeing@charteredaccountantsanz.com](mailto:cawellbeing@charteredaccountantsanz.com)
  - CPA members, please email: [countingonu@cpaaustralia.com.au](mailto:countingonu@cpaaustralia.com.au)
  - IPA members, please email: [ipacou@publicaccountants.org.au](mailto:ipacou@publicaccountants.org.au)
  - ICB members, please email: [wellbeing@icb.org.au](mailto:wellbeing@icb.org.au)
  - Registered financial planners and other business advisors, please email: counting- [on-u@deakin.edu.au](mailto:on-u@deakin.edu.au)

All information and data that has not already been used in analyses will be deleted upon your request to withdraw.

# What are the possible benefits and risks to participants?

Several key benefits are expected upon completion of this research program:

1. Business advisors will develop the skills and confidence to better support the mental health and financial wellbeing of small business owners;
2. Participants’ small business owner clients will experience improved mental health and financial wellbeing, and;
3. By developing and evaluating the training initiative, our partner organisations will be able to expand the delivery of this innovative program across the sector and on an on-going basis.

Although we believe the risks associated with your participation in this study are minimal, there are some potential consequences which should be considered. As part of your participation in the training program, you will be taught skills to help someone you may be concerned about, particularly those who may be experiencing financial distress. The

training will discuss conditions such as depression, stress, and anxiety within the context of understanding whether someone else is experiencing these conditions, and suicide will be discussed in this context. The surveys will also contain questions regarding your understanding of mental health conditions and negative feelings you might have been experiencing recently. There is the possibility that these discussions or surveys may elicit uncomfortable feelings. If this does occur, please be reminded that participation is completely voluntary, and you can withdraw at any stage. If withdrawing from the study is not sufficient to remedy these feelings, we encourage you to contact a range of helpful and free counselling services such as **Lifeline Australia (13 11 14 or www.lifeline.org.au)** and **Beyond Blue (1300 22 463 or www.beyondblue.org.au)**.

In addition to the risks outlined in this document, we recognise the challenging circumstances the COVID-19 pandemic has caused for many community members. As such, we would like to highlight that if you, or those close to you are experiencing distress, or are in need of additional support, you are encouraged to contact **Lifeline Australia (13 11 14 or www.lifeline.org.au)** and Beyond Blue **(1300 22 463 or** [**www.beyondblue.org.au**](http://www.beyondblue.org.au/)**).**

# Confidentiality and Privacy

All data collected as part of this project will be kept confidential. In the project invitation email, you were provided with an ID number. This ID number contains information about the accounting/bookkeeping body that you belong to. A correspondence table linking the ID number to your contact details (e.g. name, employer, email address) will be maintained and held by the accounting/bookkeeping body on a secure, password protected server which is only accessible to the accounting/bookkeeping body. This correspondence table will be destroyed after completion of the project. No other identifying information (e.g. name, employer, email address) will be collected with your responses.

You will be asked to consent to the research team accessing your de-identified demographic data (e.g. state, postcode, age, role) held by IPA, CPA Australia, CA ANZ or ICB. IPA, CA ANZ, and ICB members will also be asked to consent to your name and email address being provided to Deakin for the purposes of managing your registration.

You will be asked to consent to your name and email address being provided to Mentally Well Workplaces (the external vendor housing the Counting on U group of training facilitators) for the purposes of sending you Zoom session links, training materials, evaluation surveys, and for post-training coaching/mentoring follow-up. You will also be

asked to consent to your name and mailing address being provided to Mental Health First Aid Australia for the purposes of sending you a hard copy of the MHFA training manual.

These contact details will be maintained and held by Mentally Well Workplaces and

Mental Health First Aid Australia on secure, password protected servers which will only be accessible to Mentally Well Workplaces or Mental Health First Aid Australia. These records will be destroyed after completion of the project.

The research team will not collect any identifying information from peak accounting or bookkeeping bodies (e.g. name, employer, email address) unless consent is provided.

With regards to the interviews, the recording of the interview will be transcribed and any identifying information (e.g. name or organisation) will be removed, except for your ID number. In this way, the research team will only collect de-identified data. All data collected by the research team will be retained on a secure server located at Deakin University. This server is password protected, with access only available to the researchers listed, and will be retained for a period of 6 years in accordance with Deakin data security policies.

The research team will not disclose your survey responses to the accounting/bookkeeping bodies, your employer, or your small enterprise owner clients. The only information the accounting/bookkeeping bodies will have access to are membership data, consent to participate in the project, and your ID number.

It is possible that the anonymous data from this study may be shared with other researchers in the field with the aim of improving our understanding of the effectiveness of interventions aimed at improving the financial wellbeing and mental health of SME owners. Any future research project involving new researchers would need to be ethically- approved through a Human Research Ethics Committee.

# Research outputs

Your contribution to this project will result in several types of research output. The data collected as part of this study is likely to result in peer-reviewed journal articles, presentations at academic conferences and in a summary reports for interested organisations. Importantly, all the data collected as part of this research will be presented in aggregate form, meaning data will be aggregated to a group level so that individuals cannot be identified.

Project results will be provided on our website: [www.deakin.edu.au/counting-on-u.](http://www.deakin.edu.au/counting-on-u) If you would like to be emailed a copy of the results, please contact: counting-on- [u@deakin.edu.au](mailto:u@deakin.edu.au)

# Ethical research

This research is being conducted in accordance with the ethical standards statement provided by the National Health and Medical Research Council of Australia (https[://w](http://www.nhmrc.gov.au/guidelines-publications/e72))ww.[nhmrc.gov.au/guidelines-publications/e72)](http://www.nhmrc.gov.au/guidelines-publications/e72)) and has been approved by the Deakin University Human Research Ethics Committee (2020-399).

# Complaints

If you have any complaints about any aspect of the project, the way it is being conducted or any questions about your rights as a research participant, then you may contact:

The Human Research Ethics Office, Deakin University, 221 Burwood Highway, Burwood Victoria 3125, Telephone: 9251 7129, [research-ethics@deakin.edu.au](mailto:research-ethics@deakin.edu.au)

Please quote project number [2020-399]***.***
