# Supplementary material for: Counting on U training to enhance trusting relationships and mental health literacy among business advisors: protocol for a randomised controlled trial
Source: BMC Psychiatry. 2022 Jun 15;22:400. doi: 10.1186/s12888-022-04034-7 (PMC9199223; doi:10.1186/s12888-022-04034-7)
Supplement: Supplementary file 3 — Additional file 3. Additional Outcomes. [file 12888_2022_4034_MOESM3_ESM.docx]

**Supplementary File 3**

**Additional Outcomes**

*Resilience and Self-efficacy*

Psychological Capital Short Form (PCQ-12) Questionnaire measures wellbeing as a function of four emotions, two of which we are including in our analysis: resilience and self-efficacy [1]. SME owners will respond to six statements on a 6-point Likert scale (1 strongly disagree, 6 strongly agree) at baseline, 6 and 12-months follow-up and business advisors will complete these questions at 2 and 6-months follow-up. PCQ-12 is designed for working adults and both resilience and self-efficacy statements are significantly correlated with measures of satisfaction with life, happiness, and perceived stress (-0.36 to 0.50). The factor loadings of the questions range from 0.30 to 0.87.

*Social Support*

Social Support questions assess the extent of emotional social support the participant receives from clients, family and friends, or colleagues and peers [2]. The business advisors and SME owners will be asked two questions about each source: “how easy is to talk to the people concerned?” and “are the people concerned willing to listen to your problems?” Responses are recorded on a 5-point Likert scale (1 not at all, 5 a great deal). The higher the score, the higher the social support. The Cronbach’s alpha is 0.81. The SME owner will answer these questions at all time points and the business advisors at baseline and 6-months follow-up.

*Autonomy*

Autonomy will be measured using a 3-item scale that asks how much control the person feels they have over their work and scored on a 5-point Likert scale (1 strongly disagree, 5 strongly agree) ([3, 4]. A mean score will be generated and a higher score indicating greater autonomy. The Cronbach’s alpha is 0.68. Business advisors will answer these questions at baseline and 6-months follow-up.

*Emotional Demands*

Emotional Demands on the business advisors will be measured at baseline and 6-months follow-up using the second version of the Copenhagen Psychosocial Questionnaire [5]. There are four items exploring how emotionally demanding they find their work, two items are measured on one 5-point Likert scale (1 never, hardly, 5 always) and two on a different 5-point Likert scale (1 to a very small extent, 5 to a large extent). The Cronbach’s alpha is 0.87.

*Workload*

Workload of the business advisors will be measuring at baseline and 6-months follow-up using 3 items on a 5-point scale (1, not at all, 5 to a great extent) [6, 7]. They will be asked their thoughts on statements such as “to what extent is there not enough time for you to do your job?” A mean score will be generated, and a higher score indicates a more demanding workload. The Cronbach’s alpha is: 0.85 to 0.88.

*Knowledge of Mental Health*

The business advisor’s knowledge about mental health will be assessed at all times points using 18 questions adapted by Jorm et al (2010) that reviews information taught on days 1 and 2 of MHFA [8]. Questions consist of statements rated as Agree, Disagree or Unsure. The total score will be the number of questions answered correctly. An examples question is: “Half of all people who experience a mental illness have their first episode by age 18”.

*Quality of Life*

Quality of Life questions will measure the general health of the business advisors at all time points. The Short Form-12 questionnaire (SF-12) produces two summary scores – a mental component score (MCS-12) and a physical component score (PCS-12). This test has a test re-test reliability of 0.76 to 0.89 for the mental (MCS-12) and physical (PCS-12) health components [9]. Both components can discriminate among groups known to differ in their physical and mental conditions, yielding relative validities of 0.63 to 1.07 [9]. The answers are weighted, and the results are presented relative to the United States population profile at the time of the original publication in 1994. A higher score for MCS and PCS indicates a better health state.

*Help Seeking Behaviour*

Actual Help Seeking Behaviour questions will assess the business advisor’s behaviour of actively seeking assistance for any mental health problems at all time points [10]. The scale covers the informal, formal as well as physical and emotional aspects of help-seeking behaviour. The participant is asked to select from a list of people they have gone to for advice or help in the past two-weeks.

*Psychological Distress*

Psychological Distress of the business advisors will be measured using the Kessler 6 (K6) at all time points [11]. This non-specific scale screens individuals for severe mental illness as defined as a K6 score ≥13. The K6 asks respondents, in the past four weeks how often did they feel the following: nervous, hopeless, restless, or fidgety, worthless, depressed and felt that everything was an effort? For each question, a value of zero to four is assigned (0 none of the time, 4 all of the time), and the total score is summed out of 24 with a higher score indicating greater psychological distress. The scale has demonstrated excellent internal consistency and reliability (Cronbach’s alpha = 0.89) [11].

1. Kamei H, Ferreira M, Valentini F, Peres M, Kamei P, Damásio B: **Psychological Capital Questionnaire - Short Version (PCQ-12): evidence of Validity of The Brazilian Psico-USF,**. *Bragança Paulista* 2018, **23**(2):203-214.

2. Totterdell P, Wood S, Wall T: **An intra-individual test of the demands–control model: A weekly diary study of psychological strain in portfolio workers**. *Journal of Occupational and Organizational Psychology* 2006, **79**:63–84.

3. Bakker AB, Demerouti E, Verbeke W: **Using the job demands‐resources model to predict burnout and performance**. *Human Resource Management: Published in Cooperation with the School of Business Administration, The University of Michigan and in alliance with the Society of Human Resources Management* 2004, **43**(1):83-104.

4. Karasek R: **Job Content Questionnaire and User's Guide**. Lowell: University of Massachusetts, Department of Work Environment; 1985.

5. Pejtersen JH, Kristensen TS, Borg V, Bjorner JB: **The second version of the Copenhagen Psychosocial Questionnaire**. *Scandinavian journal of public health* 2010, **38**(3_suppl):8-24.

6. Albrecht K: **Stress and the Manager: Making it work for you**. New York: Simon & Schuster; 1979.

7. Karasek R: **Job demands, job decision latitude, and mental strain: Implications for job redesign**. *Administrative Science Quarterly* 1979, **24**:285-310.

8. Jorm AF, Kitchener BA, Sawyer MG, Scales H, Cvetkovski S: **Mental health first aid training for high school teachers: a cluster randomized trial**. *BMC psychiatry* 2010, **10**(1):51.

9. Ware JE, Kosinski M, Keller SD: **A 12-Item Short-Form Health Survey: Construction of Scales and Preliminary Tests of Reliability and Validity**. *Medical Care* 1996, **34**(3):220-233.

10. Rickwood D, Dean F, Wilson C, Ciarrochi J: **Young people's help-seeking for mental health problems**. *Australian e-Journal for the Advancement of Mental Health* 2005, **4**(3):1-34.

11. Kessler RC, Barker PR, Colpe LJ, Epstein JF, Gfroerer JC, Hiripi E, Howes MJ, Normand S-LT, Manderscheid RW, Walters EE: **Screening for serious mental illness in the general population**. *Archives of general psychiatry* 2003, **60**(2):184-189.
